# Dietary prebiotics and synbiotics modulate gut microbiota and improve growth performance of Mexican pike silverside *Chirostoma estor*

**DOI:** 10.7717/peerj.21435

**Published:** 2026-07-01

**Authors:** Jesús Mateo Amillano-Cisneros, Luciana Raggi, Perla T. Hernández-Rosas, Bruno Gomez-Gil, Pamela Navarrete-Ramírez, María Gisela Ríos-Durán, Carlos Cristian Martínez-Chávez, Jorge Fonseca-Madrigal, Carlos Antonio Martínez-Palacios

**Affiliations:** 1 Laboratorio Nacional de Nutrigenómica y Microbiómica Digestiva Animal (LANMDA), Instituto de Investigaciones Agropecuarias y Forestales (IIAF), Universidad Michoacana de San Nicolás de Hidalgo, Morelia, Michoacan, Mexico; 2 Laboratorio de Agroecología, Laboratorio Nacional de Innovación Ecotecnológica para la Sustentabilidad (LANIES), Instituto de Investigaciones en Ecosistemas y Sustentabilidad (IIES), Universidad Nacional Autónoma de México (UNAM), Morelia, Michoacan, Mexico; 3 Secretaría de Ciencia, Humanidades, Tecnología e Innovación (SECIHTI), Mexico City, Mexico; 4 Centro de Investigación en Alimentación y Desarrollo (CIAD), AC, Mazatlán, Sinaloa, Mexico

**Keywords:** Aquaculture biotechnology, Microbiomics, Probiotics, Aquaculture performance, Intestinal microbiota, Freshwater atherinopsidae, Agastric fish

## Abstract

Aquaculture is the fastest-growing food production sector worldwide and is vital for a sustainable animal protein supply. However, optimizing fish performance in captivity remains a major challenge, requiring functional diets that support a healthy holobiont. This study evaluated the effects of balanced experimental diets supplemented with inulin or yeast cell wall (prebiotics), *Lactobacillus acidophilus* (probiotic), or their combination (synbiotics) on juvenile pike silverside (*Chirostoma estor*). Growth performance was monitored, and gut microbiota composition was characterized by 16S rRNA gene metabarcoding. Synbiotic and yeast cell wall supplementation significantly improved growth parameters, including weight gain, final body weight, and specific growth rate, compared to the control. Microbiota profiling revealed a core community of nine genera (*Bacillus*, *Citrobacter*, *Cutibacterium*, *Lactobacillus*, *Pseudomonas*, *Spiroplasma*, *Stenotrophomonas*, *Streptococcus*, and *Thermogemmatispora*), with each treatment inducing distinct shifts in bacterial composition. Candidate probiotic taxa, including *Lactobacillus* spp., were also identified as part of the gut microbial response to dietary treatments. Functional predictions further indicated an enrichment of bacterial biosynthetic pathways in synbiotic and yeast cell wall treatments, aligning with the observed improvements in growth and feed efficiency. These findings indicated that yeast cell wall and synbiotic supplementation modulated gut microbial composition and were associated with improved growth performance in *C*.  *estor*, underscoring the role of microbiome-targeted nutrition in this species.

## Introduction

In sustainable aquaculture, the inclusion of prebiotics and probiotics either individually or combined as synbiotics has emerged as a promising strategy to enhance growth and fish health without relying on antibiotics or chemotherapeutics. Prebiotics are nondigestible dietary substrates selectively utilized by host associated microorganisms to confer health benefits, whereas probiotics are live microorganisms that improve host health by modulating the gut microbiota and its functions (FAO/WHO Nutrition Division, 2006; Gibson et al., 2017; Swanson et al., 2020). By fostering a balanced gut microbiome, these additives support essential processes such as nutrient absorption and immune competence, ultimately improving overall performance under farming conditions (Rohani et al., 2022; Sîrbu et al., 2022; Ng’onga et al., 2025).

Inulin and yeast cell wall components are common prebiotics in aquaculture, while *Lactobacillus* spp., *Bacillus* spp., and *Saccharomyces* spp. are frequently used probiotics (Wisastra et al., 2025). These treatments can be delivered through feed or the water column, depending on the species’ feeding behavior (Thakur et al., 2025). Research in teleosts indicates that these functional ingredients improve growth and survival by modulating the intestinal microbiota and host metabolism (Nayak, 2010; Wisastra et al., 2025). Therefore, the gut microbiome has become a sensitive indicator of physiological condition and a key target for dietary intervention.

Despite the demonstrated benefits of these supplements in various teleost species, their effects remain largely unexplored in agastric digestive models with a short intestine such as the pike silverside (*Chirostoma estor*). This species possesses a highly dynamic gut microbiota and a defined core community that likely facilitates efficient nutrient assimilation within its simplified digestive configuration (Amillano-Cisneros et al., 2022). These characteristics make *C. estor* an ideal reductionist model for investigating how targeted dietary interventions influence gut microbial communities and host performance.

Atherinopsids or silversides comprise approximately 70 species inhabiting freshwater, estuarine, and marine environments globally (Miller, Minckley & Norris, 2005). These fish are notable for their high concentrations of omega 3 polyunsaturated fatty acids, particularly DHA, which far exceed the levels found in other freshwater teleosts like cyprinids and cichlids (Martínez-Palacios et al., 2020). This superior fatty acid profile confers exceptional nutritional value and positions atherinopsids as outstanding freshwater fish capable of providing high quality proteins and lipids for human consumption.

In this study, we evaluated the effects of dietary prebiotics, probiotics, and synbiotics on the growth, survival, fatty acid composition, and gut microbiota of juvenile *C. estor.* We further propose this species as a novel agastric model to disentangle the complex interactions between diet, the microbiome, and the host. These outcomes are expected to deepen our understanding of holobiont functioning in simplified digestive systems and support the development of sustainable, microbiome informed culture strategies for *C. estor* and other aquaculture species.

## Materials and Methods

All procedures were conducted in accordance with Mexican federal regulations for laboratory animal care (NOM-062-ZOO-1999) and EU Directives 2010/63/EU and 2007/526/EC, and were approved by the Universidad Michoacana de San Nicolás de Hidalgo (CEIIB 254-2025) and SECIHTI (315841).

### Fish farming and collection

All fish used in this study were obtained from the Laboratorio Nacional de Nutrigenómica, Microbiómica y Digestiva Animal (LANMDA), Instituto de Investigaciones Agropecuarias y Forestales, Universidad Michoacana de San Nicolás de Hidalgo, Morelia, Mexico (19°41′22″N, 101°14′56″W; 23 °C; 1,896 m a.s.l.), operating under an ISO 9001:2015 quality management system. At this facility, *Chirostoma estor* has been maintained in freshwater aquaculture under a closed life cycle for more than 20 years, with fish routinely reared through five cultured stages from egg to adult. In autumn 2018, seven-month-old juvenile *C. estor* were collected from indoor stock tanks and transferred to experimental units.

### Potential prebiotics and probiotics supplemented diets

Potential prebiotics, inulin and yeast cell wall were acquired from local commercial distributors. Inulin from *Agave tequilana* (Transformación e Innovación S.A. de C.V. Jalisco, Mexico), and *Saccharomyces cerevisiae* cell wall, containing mannan-oligosaccharides (MOS, ≥22%) and *β*-glucans (≥24%) (SafMannan^®^, Safmex S.A. de C.V. Lima, Peru). The potential probiotic bacteria, *Lactobacillus acidophilus* La-14^®^ (Danisco Inc., Copenhagen, Denmark) were cultured in a MRS broth (Difco™) at 37 °C for 48 h, centrifuged, and washed with sterile distilled water before adding to the mix for pellet preparation (see Table S1 for ingredient quantities). The *L*. *acidophilus* final density, quantified in MRS agar (Difco™), was 10^6^ CFU/g in all supplemented feeds; inulin and cell wall were added in the following concentrations, respectively, 20 g kg^−1^ and 0.25 g kg^−1^. Six experimental pelleted diets (41% protein) were formulated and produced in our laboratory: (1) a basal, unsupplemented control diet (C); (2) basal diet supplemented with *Lactobacillus acidophilus* (L); (3) basal diet supplemented with inulin (I); (4) basal diet supplemented with a prebiotic cell wall preparation (W); (5) basal diet supplemented with synbiotics *L. acidophilus* + inulin (L+I); and (6) basal diet supplemented with synbiotics *L. acidophilus* + cell wall (L+W) (Table S1). For pellet preparation, all ingredients for each diet were thoroughly mixed and passed through a Hobart meat grinder to produce pellets (four mm diameter). Pellets were dried in a forced-air dryer at 25–30 °C for 24 h until a moisture content of approximately 10% was reached, after which they were broken into smaller pellets (1–2 mm) suitable for feeding.

### Experimental design

A total of 2,034 juvenile *Chirostoma estor* (mean body weight 5.6 ± 1.8 g; total length 9.2 ± 1.0 cm, mean ± SD) were randomly stocked into 18 circular tanks (7,000 L; 3 m diameter × 1 m height) within an open-flow freshwater (ground water) aquaculture system. Tanks were continuously aerated and operated under an open-flow system with a water exchange rate of seven L min^−1^. Tanks were randomly assigned to six dietary treatments, initially with three replicate tanks per treatment (113 fish per tank), and spatially distributed within the facility to minimize environmental confounding. Fish were acclimated for two weeks and fed a basal diet prior to the start of the experiment. The feeding trial lasted 12 weeks. Fish were fed their respective experimental diets manually to apparent satiation six times per day over a 9 h feeding period. Water temperature and dissolved oxygen were monitored daily and maintained at 19.6 ± 1.8 °C and 4.01 ± 0.62 mg L^−1^, respectively.

At the end of the experiment, 20 fish were randomly sampled from each replicate tank two hours post-feeding to assess growth and morphometric parameters. From these individuals, a subset of three fish per replicate tank was randomly selected for gut microbiota and fatty acid analyses. These three fish per replicate tank were used as biological subsamples for gut microbiome analysis (nine individuals per treatment). Sampled fish were immediately anesthetized using cold water and euthanized. Each fish was measured and weighed individually, and the entire gastrointestinal tract was aseptically excised from the posterior esophagus to the anus, placed individually in sterile tubes containing 96% ethanol, and stored at 4 °C until DNA extraction. Remaining fish were returned to holding tanks at the aquaculture biotechnology facility.

### Proximate composition analysis

Proximate analyses of diets and fish were performed in triplicate following standard protocols (AOAC, 2000). Moisture content was determined according to procedure 934.01 by drying samples at 100 °C for 24 h in a Fisher Scientific oven (Waltham, MA, USA). Crude fat was analyzed *via* method 920.39 using ether extraction with a Foss Tecator 2050 Soxtec Avanti auto extraction unit (Cobham, England). Crude protein was measured using the Dumas method (Ebling, 1968) following procedure 968.06 (N x 6.25) with a Leco FP 528 nitrogen analyzer (St. Joseph, MI, USA). Finally, crude ash was determined through method 942.05 by incineration at 600 °C in a Fisher Scientific muffle furnace (MA, Waltham, MA, USA).

### Growth performance

After 12 weeks fish performance was evaluated in terms of final body length (FBL), final body weight (FBW), weight gain (WG), weight gain rate (WGR), specific growth rate (SGR), condition factor (CF), feed intake (FI), feed conversion ratio (FCR), feed efficiency (FE), and survival rate (SR).

Indices were calculated as follows:

WG (g) = (final mean body weight - initial mean body weight)

WGR (%) = 100 x [(final mean body weight - initial mean body weight)/initial mean body weight)]

SGR (% day^−1^) = 100 x [(ln (final body weight)–ln (initial body weight)/days]

CF = 100 × (weight, g)/(body length, cm)^3^

FCR = feed intake (g)/weight gain (g)

FE = weight gain (g)/feed intake (g)

SR = 100 × (final number of animals)/(initial number of animals)

### Fatty acid analysis

Saturated (SFA), monounsaturated (MUFA), polyunsaturated fatty acids (PUFA), and long-chain polyunsaturated fatty acids (LC-PUFA) profiles were determined from eviscerated fish from all treatments following (Martínez-Palacios et al., 2020). Lipids were extracted using a Foss Tecator Soxhlet extraction system (Soxtec 2050, Avanti). Fatty acid methyl esters (FAME) were prepared by acid-catalyzed transesterification using 14% boron trifluoride in methanol and analyzed by gas chromatography using an Agilent Technologies GC 6850 equipped with a DB-23 silica column and flame ionization detector.

### DNA sequencing and bioinformatic analysis

Metagenomic DNA extraction, sequencing, and bioinformatic analyses were performed as described by Amillano-Cisneros et al. (2022). Briefly, DNA was extracted from 30–50 mg of fish gut tissue using the CTAB method adapted from Doyle & Doyle (1987). The V3 region of the 16S rRNA gene was amplified by PCR using primers V3-338f and V3-533r (Huse et al., 2008). For indexation, the Nextera XT v2 dual-index barcodes were used. The PCR program consisted of a single cycle at 72 °C for 3 min, followed by 14 cycles of 95 °C for 30 s, 55 °C for 30 s, and 72 °C for 15 s, with a final extension at 72 °C for 5 min. Sequencing was performed on an Illumina MiniSeq platform using a mid-output flow-cell with 300 cycles (2 × 150 bp) at CIAD, Mazatlán, Sinaloa, Mexico. Sequence data are available under BioProject accession PRJNA796804 (SRA accessions SAMN25047428 –SAMN25047469).

Raw reads were quality-checked, trimmed, and assembled using PRINSEQ (Schmieder & Edwards, 2011). Sequences were dereplicated, chimeras were removed, and the remaining reads were clustered into OTUs at 97% similarity using VSEARCH (Rognes et al., 2016). Taxonomic assignment was performed against the SILVA 138 database (Quast et al., 2013). The phyloseq R package (McMurdie & Holmes, 2013) was used to calculate diversity metrics using the Bray–Curtis dissimilarity index. Visualization and statistical analyses were conducted using the vegan package v2.5-4 (Oksanen et al., 2019) in R (R Core Team, 2013) to assess differences among treatments.

For differential genus analysis, linear discriminant analysis (LDA) effect size (LEfSe) analysis was applied to identify bacterial taxa with significant differences in abundance between treatments (Segata et al., 2011). LEfSe was performed using abundance data at the phylum and genus levels across the six treatments within the Galaxy framework. Differentially abundant taxa were identified using the non-parametric Kruskal–Wallis test, with LDA scores representing the estimated effect size for each taxon within individual pairwise treatment comparisons. Pairwise comparison outputs (*e.g.*, control *vs.* Lactobacillus, control *vs.* inulin, and remaining treatment combinations) were visualized using LDA scores (log10) generated with a custom R script. Results were summarized by grouping treatments according to the taxa identified as differentially abundant in each comparison.

For functional analysis, hidden-state prediction was performed using Phylogenetic Investigation of Communities by Reconstruction of Unobserved States 2 (PICRUSt2) to infer gene family abundances, employing the castor R package. Pathway abundances were calculated using the Kyoto Encyclopedia of Genes and Genomes (KEGG) database *via* the pathway_pipeline.py script. The resulting output file (path_abun_unstrat_descrip.tsv), containing predicted pathway abundances, was imported into R (R Core Team, 2013).

A heatmap representing the overall average across samples was generated using the ComplexHeatmap package (Gu, Eils & Schlesner, 2016) to visualize the 60 most abundant functional pathways. Additionally, the PICRUSt2 output file was loaded into MEGAN5 (Huson et al., 2016) to assign metabolic categories. Differences among the six treatment groups were assessed using a two-sided Welch’s *t*-test, with *p* < 0.05 considered statistically significant. Results were visualized as heatmaps generated in R.

### Statistical analysis

All statistical analyses were performed in R version 4.4.2 (R Core Team, 2013). Growth performance variables (final body length, final body weight, weight gain, weight gain rate, specific growth rate, condition factor, feed intake, feed conversion ratio, feed efficiency, and survival) were first evaluated for normality and homogeneity of variance. Because at least one assumption was violated for all variables, differences among dietary treatments were assessed using the non-parametric Kruskal–Wallis test, followed by Dunn’s *post hoc* comparisons with Benjamini–Hochberg correction for multiple testing.

The sample size for metagenomic barcoding analysis was determined using gut microbiota community composition (represented by a Bray–Curtis dissimilarity matrix) as the primary dependent variable. We considered a standard alpha error of 0.05. Based on our previous study of *C*. *estor* (Amillano-Cisneros et al., 2022), which revealed that external factors significantly explained variations in microbiota composition (*R*^2^ = 0.877, *p* = 0.001), we determined that nine individuals per treatment provided sufficient sensitivity to detect dietary effects. This sampling intensity is supported by the framework of Kelly et al. (2015), which indicates that sample sizes of 5–10 subjects per group typically provide high statistical power (often reaching 90%) for detecting differences in microbiome studies analyzed *via* PERMANOVA. This approach balances biological representation with the ethical Principle of Reduction to minimize animal use, in accordance with EU Directives 2010/63/EU.

To account for the hierarchical experimental design and the reduced replication of one dietary treatment (Lactobacillus; L), growth data were additionally analyzed using generalized linear mixed-effects models (GLMMs). Models were fitted at the individual level using final body length or final body weight as response variables, dietary treatment and standard length as fixed effects, and tank and individual identity as random effects. Models were fitted using maximum likelihood with Laplace approximation. Analyses conducted with and without the L treatment yielded qualitatively consistent results (results not shown). *Post hoc* contrasts were performed using estimated marginal means implemented in the *emmeans* R package.

Fatty acid data were evaluated for normality and homogeneity of variance and subsequently analyzed using one-way analysis of variance (ANOVA). Differences in gut microbiota alpha diversity (Shannon index) among treatments were assessed using the non-parametric Kruskal–Wallis test followed by Dunn’s *post hoc* test with Benjamini–Hochberg correction. Relationships between alpha diversity and growth performance were evaluated using Spearman’s rank correlation. Canonical correspondence analysis (CCA) was used to examine associations between gut microbiota composition and growth performance. Statistical significance was set at *α* = 0.05.

## Results

### Fish performance

Pike silverside fed synbiotic diets (*L*. *acidophilus* + inulin [L+I] and *L*. *acidophilus* + yeast cell wall [L+W]) exhibited the highest final body weight (FBW) and final body length (FBL). Global Kruskal–Wallis tests indicated significant dietary effects on FBW, FBL, weight gain (WG), specific growth rate (SGR), feed efficiency (FE), and feed conversion ratio (FCR) (*p* < 0.001 for all variables). *Post hoc* pairwise comparisons using Dunn’s test with Benjamini–Hochberg correction revealed that FBW, FBL, WG, SGR, and FE were significantly higher in L+I, L+W, and W diets compared with control (C), probiotic-only (L), and prebiotic-only (I) diets (*p* < 0.05), whereas differences among L+I, L+W, and W were smaller but remained significant for some variables (*e.g.*, FBW: L+I *vs.* W, *p* = 0.0429). Feed conversion ratio was lowest in synbiotic and W treatments (1.21–1.46), indicating superior feed utilization relative to other diets (2.42–3.23). Survival rates were high across all treatments (98.23–99.82%). Two replicate tanks in the *L* treatment experienced early mortalities within the first two weeks; therefore, survival was estimated using the remaining tank (Table 1).

**Table 1:** Zootechnical performance parameters. Mean values and standard deviations of proximate carcass composition (% on a wet matter basis ± SD) of pike silverside *C. estor*, fed with control (C), probiotic (*L. acidophilus*, L), prebiotics (inulin, I; cell wall of *S. cerevisiae*, W) and synbiotics (*L. acidophilus* + inulin, L+I; *L. acidophilus* + cell wall, L+W) diets.

	**Treatments**						
	**Control (C)** **(*n* = 60)**	***L*** **.** ***acidophilus*** ** (L )** **(*n* = 20)**	**Inulin (I)** **(*n* = 60)**	**Yeast cell wall (W)** **(*n* = 60)**	**L+I** **(*n* = 60)**	**L+W** **(*n* = 60)**	*P*-value
IBL (cm)	9.20 ± 1.00	9.20 ± 1.00	9.20 ± 1.00	9.20 ± 1.00	9.20 ± 1.00	9.20 ± 1.00	N/A
IBW (g)	5.60 ± 1.80	5.60 ± 1.80	5.60 ± 1.80	5.60 ± 1.80	5.60 ± 1.80	5.60 ± 1.80	N/A
FBL (cm)	12.03 ± 0.81**c**	11.78 ± 0.91**c**	12.11 ± 0.93**c**	12.88 ± 0.78**b**	13.59 ± 0.92**a**	13.59 ± 0.68**a**	2.2e−16
FBW (g)	11.76 ± 2.78**c**	11.57 ± 3.06**c**	12.02 ± 2.86**c**	15.44 ± 3.07**b**	17.68 ± 2.49**a**	16.91 ± 3.49**a**	2.2e−16
WG (g)	6.14 ± 2.78**c**	5.95 ± 3.06**c**	6.40 ± 2.86**c**	9.81 ± 3.07**b**	12.06 ± 2.49**a**	11.29 ± 3.49**a**	2.2e−16
WG (%)	109.16 ± 49.47**c**	105.78 ± 54.40**c**	113.88 ± 50.86**c**	174.64 ± 54.67**b**	214.59 ± 44.36**a**	200.86 ± 62.09**a**	2.2e−16
SGR	0.85 ± 0.26**c**	0.82 ± 0.30**c**	0.87 ± 0.27**c**	1.18 ± 0.23**b**	1.35 ± 0.17**a**	1.29 ± 0.23**a**	2.2e−16
CF	96.72 ± 16.10**c**	97.04 ± 17.96**c**	98.47 ± 17.21**c**	118.94 ± 16.75**b**	129.82 ± 13.94**a**	123.60 ± 19.50**b**	2.2e−16
FI	12.23 ± 0.48	14.23 ± 0.00	13.24 ± 0.65	13.04 ± 0.79	14.71 ± 2.61	12.61 ± 1.38	0.08585
FCR	2.42 ± 1.17**a**	3.23 ± 2.55 **a**	2.49 ± 1.13 **a**	1.46 ± 0.46**b**	1.28 ± 0.36**c**	1.21 ± 0.33**c**	2.2e−16
FE	0.50 ± 0.22**c**	0.42 ± 0.21**c**	0.48 ± 0.22**c**	0.75 ± 0.23**b**	0.84 ± 0.21**a**	0.89 ± 0.25**a**	2.2e−16
SR (%)	98.82 ± 1.35	99.12 ± 0.00	98.23 ± 0.88	99.12 ± 0.88	98.82 ± 0.51	99.41 ± 0.51	0.5568
Proximate composition (% ± SD)							
	*n* = 3	*n* = 3	*n* = 3	*n* = 3	*n* = 3	*n* = 3	
Moisture	76.95 ± 0.47	79.11 ± 1.38	77.36 ± 0.09	76.57 ± 0.74	78.28 ± 0.77	76.97 ± 1.25	0.0361
Ash	2.88 ± 0.19	2.61 ± 0.12	2.8 ± 0.15	2.57 ± 0.06	2.77 ± 0.10	2.68 ± 0.18	0.118
Lipid	4.74 ± 0.10**b**	3.72 ± 0.03**b**	4.66 ± 0.23**b**	5.84 ± 0.09**a**	4.5 ± 0.05**b**	4.47 ± 0.29**b**	9.58e−08
Protein	16.11 ± 0.55**a**	14.84 ± 0.27**b**	15.03 ± 0.17**b**	15.23 ± 0.07**b**	15.09 ± 0.18**b**	15.58 ± 0.08**a**	0.00115

**Notes.**

Statistically significant differences between rows (*p* < 0.05) are indicated by different letters.

L+I *L*. *acidophilus* + Inulin L+W *L*. *acidophilus* + Yeast cell wall IBL initial body length IBW initial body weight FBL final body length FBW final body weight WG weight gain in grams WG weight gain (%) SGR specific growth rate CF condition factor FI feed intake FCR feed conversion ratio FE feed efficiency SR survival rate

GLMM analyses (Bolker et al., 2009), accounting for tank and individual effects, corroborated these findings: treatment estimates were significantly lower for C, L, and I diets compared with the reference diet (control) for both FBW and FBL (*e.g.*, W: estimate = −0.021 ±  0.003, t = −7.72, *p* < 0.001; L+I: estimate = −0.031 ± 0.003, t = −11.38, *p* < 0.001; L+W: estimate = −0.028 ± 0.003, t = −10.01, *p* < 0.001). These combined results demonstrate robust diet-related growth differences.

Proximate composition of the fish showed minor differences among treatments. Moisture and ash content were similar, whereas lipid content was slightly higher in W-fed fish (5.84 ± 0.09%), and protein content was highest in L+W-fed fish (15.58 ±  0.08%) (Table S1). Fatty acid profiles were largely unaffected (Table S2).

### Gut microbiota diversity and composition

**Figure 1:**
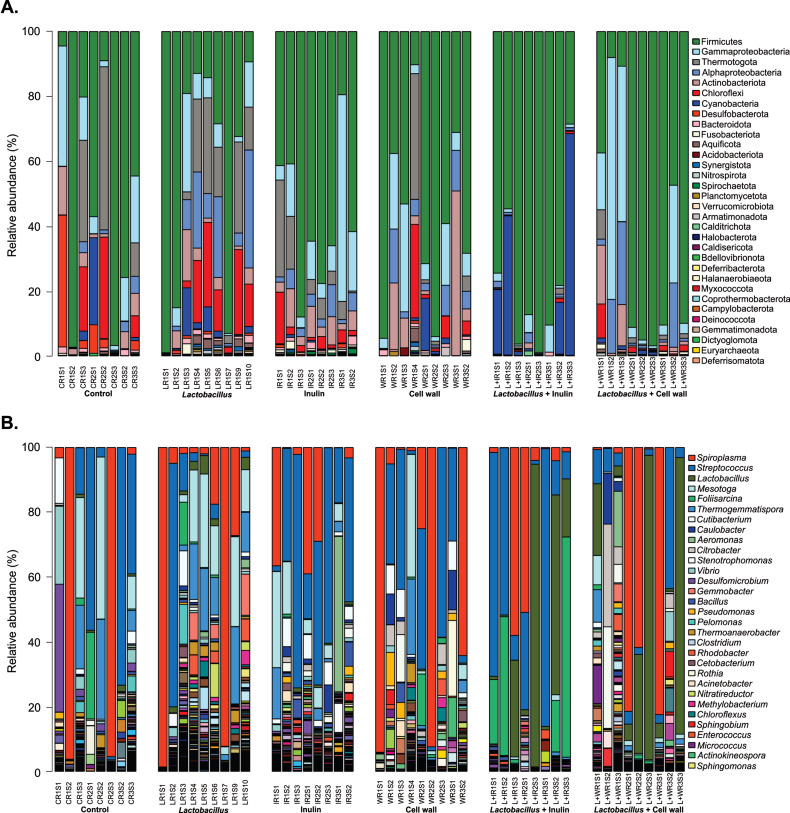
Relative taxonomic abundance of pike silverside gut microbiota. Fish were fed control, probiotic (*Lactobacillus acidophilus*), prebiotic (inulin or cell wall), and synbiotic (*L*. *acidophilus* + inulin; *L*. *acidophilus* + cell wall) diets. Microbial composition is shown at the (A) phylum and (B) genus levels.

At the phylum level, Firmicutes (currently Bacillota) was dominant (57.95 ± 4.48%), followed by Proteobacteria (17.33 ± 3.23%), Thermotogota (6.60 ± 1.64%), Chloroflexi (4.94 ± 1.18%), Actinobacteriota (4.86 ± 1.16%), and Cyanobacteriota (4.67 ± 1.70%) (Fig. 1A and Table S3). At the genus level, *Spiroplasma* (23.27 ± 4.83%), *Streptococcus* (22.05 ± 3.58%), and *Lactobacillus* (9.43 ± 3.25%) were most abundant (Fig. 1B; Table 2).

**Figure 2:**
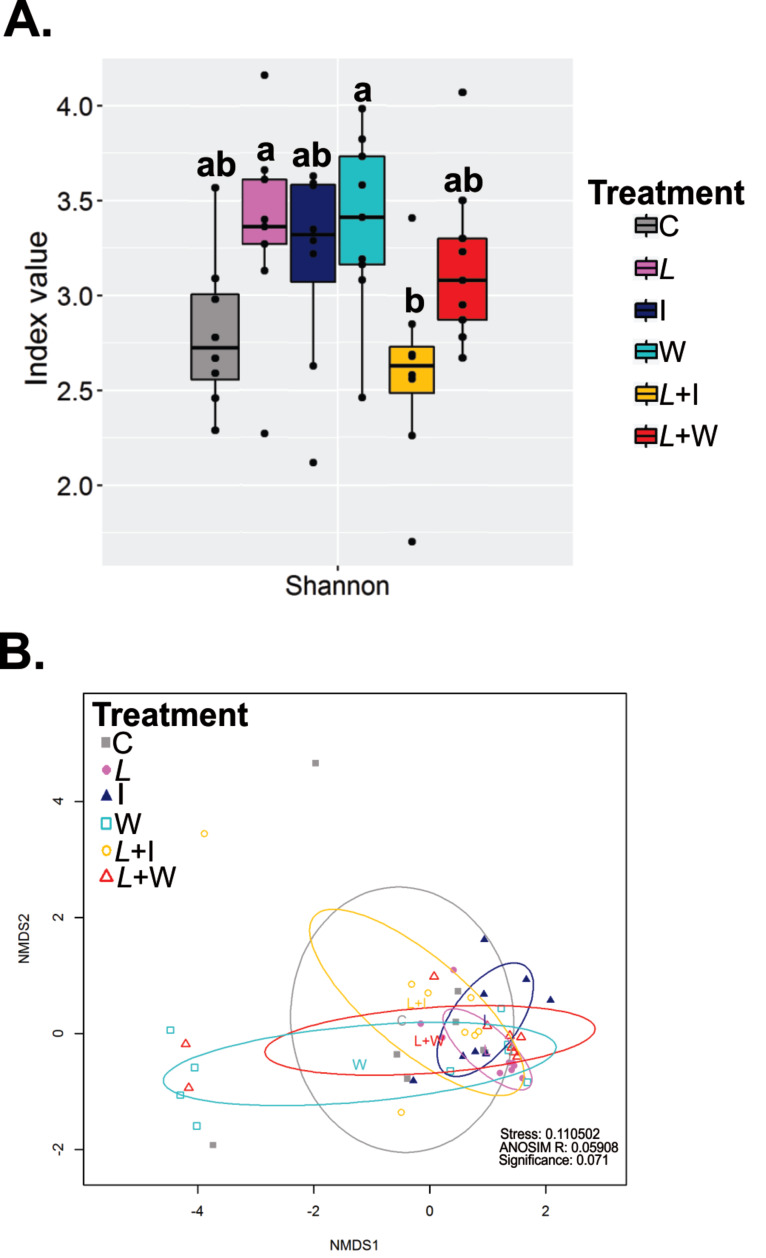
Alpha and beta diversity of the gut bacterial community. (A) Alpha diversity (richness Shannon diversity index) (B) and beta diversity based on non-metric multidimensional scaling (NMDS) analysis were calculated using OTUs data. Treatments are shown in the legend: control (basal diet, C); supplemented with probiotic (*Lactobacillus acidophilus*, L), prebiotic (inulin, I; cell wall, W), and synbiotics (*L. acidophilus* + inulin, L+I; *L. acidophilus* + cell wall, L+W).

Alpha diversity (Shannon index) was highest in L and W diets and lowest in L+I (Kruskal–Wallis *p* = 0.01322; Dunn-BH *post hoc*; Table S4), whereas beta diversity (NMDS) revealed partial clustering among treatments, with significant differences detected between L *vs.* W, L *vs.* L+I, I *vs.* L+I, and W *vs.* L+I (PERMANOVA, *p* < 0.05) (Fig. 2; Table S5).

**Figure 3:**
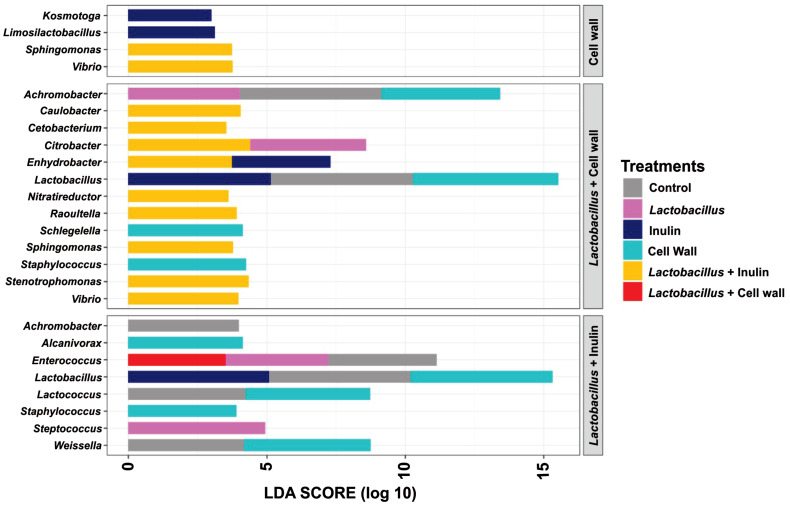
Gut microbiota differential bacterial genera within the different treatments. Linear Discriminative Analysis (LDA) shows differentially abundant genera in each treatment (gray box) when compared to treatments in legend. When there are no differences, no color bar is generated. See Figs. S7 –S9 for Control, Inulin, and *Lactobacillus* treatments.

**Figure 4:**
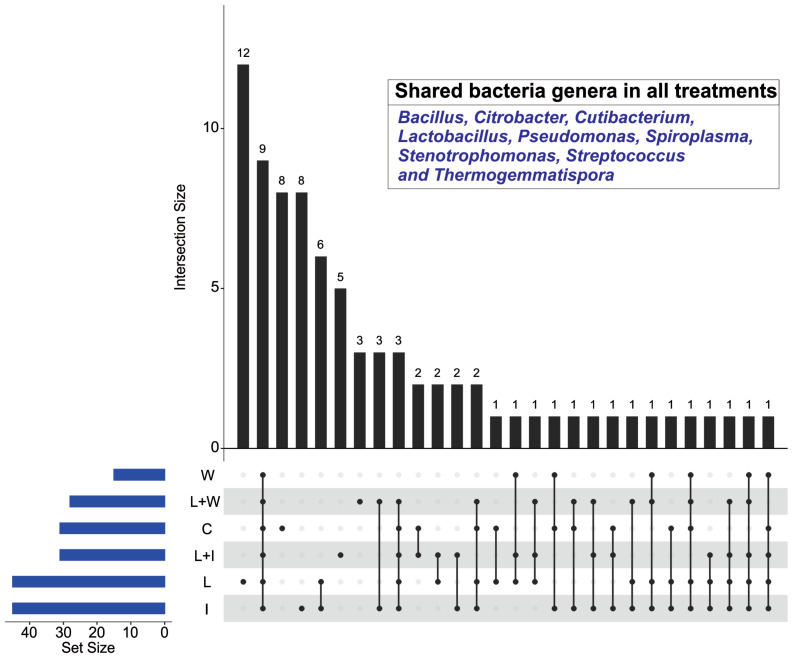
UpSet plot showing shared bacterial genera across treatments. Treatments include control (C), *Lactobacillus acidophilus* (L), inulin (I), cell wall (W), *L*. *acidophilus* + inulin (L+I), and *L*. *acidophilus* + cell wall (L+W).

LEfSe analysis identified differentially abundant genera among treatments, with *L. acidophilus*–containing diets enriched in *Lactobacillus*, while the synbiotic and W diets exhibited distinct shifts in several genera, including *Kosmotoga, Limosilactobacillus, Achromobacter,* and *Sphingomonas* (Fig. 3; Table S5). Core microbiota, present in ≥80% of samples, included *Bacillus, Citrobacter, Cutibacterium, Lactobacillus, Pseudomonas, Spiroplasma, Stenotrophomonas, Streptococcus,* and *Thermogemmatispora* (Fig. 4; Table S6).

**Table 2:** Relative abundance of bacterial genera gut community. Total mean (%) relative abundance (second column) ± SE, and mean relative abundance ± SE of the 30 genus prevalent found in gut samples of pike silverside fed with experimental diets.

	**Total abundance**	**Treatments**						
**Genus**		**C**	**L**	**I**	**W**	**L+I**	**L+W**	
	**(*N* = 51)**	**(*N* = 8)**	**(*N* = 9)**	**(*N* = 8)**	**(*N* = 9)**	**(*N* = 8)**	**(*N* = 9)**	*P*-value
*Spiroplasma*	23.27 ± 4.83	24.65 ± 15.56	27.44 ± 13.21	13.80 ± 6.26	31.42 ± 13.60	15.05 ± 8.66	25.43 ± 12.62	0.7029
*Streptococcus*	22.05 ± 3.58	23.13 ± 10.25**ab**	10.79 ± 8.15**b**	36.09 ± 8.52**a**	23.31 ± 7.53**ab**	33.07 ± 11.36**a**	8.81 ± 4.44**b**	0.09862
*Lactobacillus*	9.43 ± 3.25	0.54 ± 0.24**bc**	1.96 ± 0.64**b**	0.53 ± 0.15**b**	0.18 ± 0.06**c**	25.92 ± 12.26**a**	27.31 ± 12.32**a**	0.00057
*Mesotoga*	6.52 ± 1.62	11.47 ± 6.70**ab**	12.45 ± 3.94**ab**	8.34 ± 3.53**a**	4.96 ± 4.14**ab**	0.35 ± 0.23**b**	1.60 ± 0.96**ab**	0.02365
*Foliisarcina*	4.29 ± 1.70	3.75 ± 3.31	1.69 ± 1.44**b**	0.21 ± 0.10**b**	2.18 ± 1.72**b**	18.51 ± 8.85**a**	0.45 ± 0.20**b**	0.6263
*Thermogemmatispora*	4.24 ± 1.07	7.13 ± 4.16**a**	9.75 ± 3.05**a**	3.04 ± 1.89**ab**	3.24 ± 2.54**ab**	0.33 ± 0.14**b**	1.73 ± 1.08**b**	0.1566
*Cutibacterium*	2.12 ± 0.48	2.57 ± 1.71**ab**	2.06 ± 1.18**ab**	4.05 ± 1.18**a**	3.29 ± 1.42**ab**	0.21 ± 0.09**b**	0.61 ± 0.17**b**	0.09569
*Aeromonas*	1.53 ± 0.99	0.11 ± 0.08**b**	0.60 ± 0.31**a**	6.17 ± 5.98**a**	0.27 ± 0.19**ab**	0.05 ± 0.02**b**	2.15 ± 1.87**ab**	0.2133
*Citrobacter*	1.50 ± 0.65	0.28 ± 0.11**b**	0.18 ± 0.07**b**	0.83 ± 0.27**b**	2.26 ± 0.91**a**	0.09 ± 0.05**b**	5.00 ± 3.41**a**	0.02812
*Stenotrophomonas*	1.40 ± 0.67	0.35 ± 0.19**ab**	0.39 ± 0.12**ab**	1.24 ± 0.45**a**	1.79 ± 1.61**ab**	0.01 ± 0.00**b**	4.35 ± 3.43**a**	0.01985
*Caulobacter*	1.28 ± 0.44	0.13 ± 0.07**b**	0.46 ± 0.15**ab**	1.94 ± 1.01**a**	2.68 ± 1.56**a**	0.09 ± 0.06**b**	2.20 ± 1.72**a**	0.0136
*Vibrio*	1.23 ± 0.51	4.17 ± 2.92**a**	0.87 ± 0.29**b**	0.69 ± 0.25**a**	0.65 ± 0.26**a**	0.02 ± 0.01**b**	1.13 ± 1.01**a**	0.02991
*Desulfomicrobium*	0.97 ± 0.78	5.86 ± 4.83**a**	0.12 ± 0.10**bc**	0.16 ± 0.10**b**	0.00 ± 0.00**c**	0.05 ± 0.02**b**	0.00 ± 0.00**c**	0.001333
*Bacillus*	0.70 ± 0.21	0.36 ± 0.17**b**	0.39 ± 0.12**b**	2.16 ± 0.99**a**	0.84 ± 0.67**b**	0.39 ± 0.16**ab**	0.15 ± 0.04**b**	0.06067
*Gemmobacter*	0.68 ± 0.32	0.02 ± 0.01**c**	2.94 ± 1.55**ab**	0.09 ± 0.03**ab**	0.04 ± 0.02**c**	0.15 ± 0.14**bc**	0.62 ± 0.61**bc**	0.03913
*Pseudomonas*	0.68 ± 0.23	0.37 ± 0.25**ab**	0.13 ± 0.07**b**	0.76 ± 0.36**a**	2.24 ± 1.15**ab**	0.03 ± 0.01**b**	0.46 ± 0.26**ab**	0.1971
*Pelomonas*	0.66 ± 0.26	1.20 ± 0.67**a**	1.46 ± 1.36**a**	0.44 ± 0.12**a**	0.19 ± 0.10**ab**	0.06 ± 0.02**b**	0.57 ± 0.24**ab**	0.09163
*Thermoanaerobacter*	0.65 ± 0.19	0.69 ± 0.51**a**	1.66 ± 0.59**a**	0.40 ± 0.19**a**	0.75 ± 0.66**a**	0.05 ± 0.03**b**	0.29 ± 0.16**a**	0.2124
*Rhodobacter*	0.55 ± 0.23	0.00 ± 0.00**b**	1.94 ± 0.94**a**	0.01 ± 0.01**b**	0.60 ± 0.57**a**	0.01 ± 0.00**b**	0.57 ± 0.56**a**	0.007744
*Rothia*	0.49 ± 0.46	0.00 ± 0.00**ab**	0.02 ± 0.02**ab**	0.05 ± 0.04**ab**	2.69 ± 2.59**a**	0.00 ± 0.00**b**	0.00 ± 0.00**ab**	0.1944
*Acinetobacter*	0.48 ± 0.13	0.36 ± 0.19	0.36 ± 0.15	0.88 ± 0.50	0.75 ± 0.54	0.09 ± 0.04	0.41 ± 0.22	0.8852
*Cetobacterium*	0.41 ± 0.10	0.15 ± 0.08**bc**	0.45 ± 0.35**ab**	0.80 ± 0.25**a**	0.51 ± 0.37**b**	0.05 ± 0.03**c**	0.46 ± 0.17**ab**	0.03655
*Nitratireductor*	0.40 ± 0.21	0.09 ± 0.05**bc**	1.64 ± 1.13**a**	0.23 ± 0.08**a**	0.22 ± 0.19**abc**	0.02 ± 0.01**c**	0.13 ± 0.04**ab**	0.01971
*Chloroflexus*	0.39 ± 0.15	0.09 ± 0.04**b**	1.07 ± 0.52**a**	0.07 ± 0.03**b**	0.95 ± 0.60**a**	0.00 ± 0.00**c**	0.04 ± 0.02**bc**	0.04175
*Sphingobium*	0.37 ± 0.18	0.04 ± 0.02**b**	0.14 ± 0.05**a**	0.40 ± 0.29**a**	0.60 ± 0.49**a**	0.01 ± 0.01**b**	0.93 ± 0.86**a**	0.4704
*Methylobacterium*	0.35 ± 0.11	0.12 ± 0.06	1.18 ± 0.44	0.11 ± 0.07	0.44 ± 0.34	0.10 ± 0.08	0.04 ± 0.02	0.01286
*Micrococcus*	0.34 ± 0.24	0.05 ± 0.04**ab**	0.02 ± 0.01**ab**	0.07 ± 0.02**a**	0.33 ± 0.29**ab**	0.00 ± 0.00**b**	1.45 ± 1.30**ab**	0.2553
*Actinokineospora*	0.33 ± 0.24	0.03 ± 0.03**ab**	0.07 ± 0.04**ab**	0.30 ± 0.13**a**	1.40 ± 1.35**ab**	0.00 ± 0.00**b**	0.08 ± 0.04**ab**	0.2444
*Enterococcus*	0.31 ± 0.07	0.12 ± 0.05**c**	0.06 ± 0.04**c**	0.52 ± 0.16**ab**	0.30 ± 0.16**abc**	0.80 ± 0.32**a**	0.14 ± 0.05**c**	0.00975
*Clostridium*	0.10 ± 0.02	0.12 ± 0.06	0.11 ± 0.04	0.11 ± 0.04	0.04 ± 0.02	0.09 ± 0.03	0.12 ± 0.07	0.5915

**Notes.**

C Control L Lactobacillus I inulin W yeast cell wall L+I L. acidophilus + inulin W L. acidophilus + cell wall

Different letters per row indicate statistical significance between treatments abundances (*p* < 0.05).

Spearman rank correlation analyses revealed no significant associations between gut microbial alpha diversity and growth performance. Shannon index was weakly negatively correlated with final body weight (*ρ* = −0.37, *p* = 0.47), indicating that alpha diversity did not directly predict growth under the experimental conditions. Additional correlations between other growth variables (WG, SGR, FCR, FE) and microbial taxa were not significant after Benjamini–Hochberg correction (results not shown).

**Figure 5:**
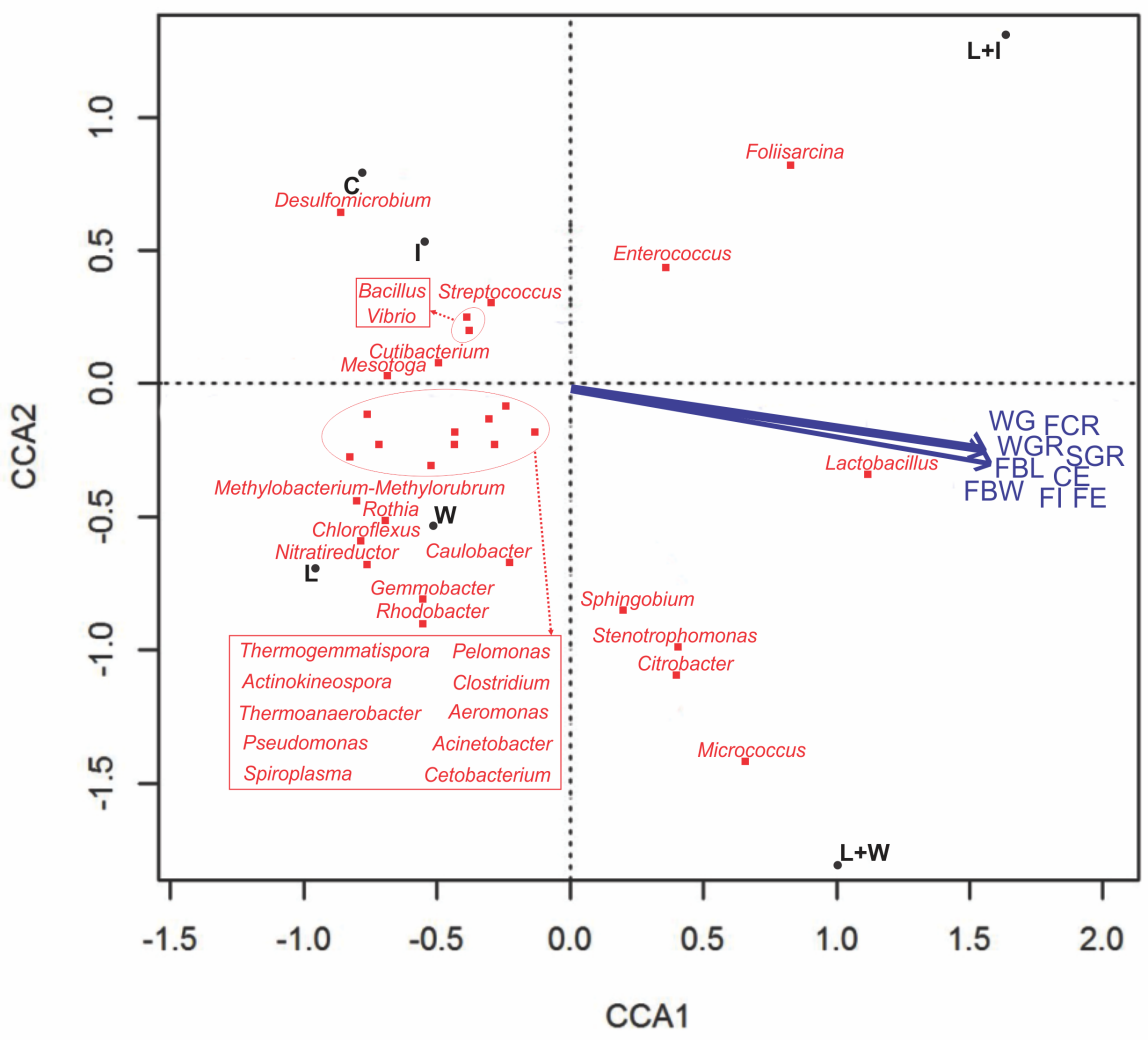
Canonical Correspondence Analysis (CCA) of microbiota genera and fish growth parameters. Blue labels represent growth parameters. Red labels represent the 30 most abundant genera across treatments. Black capital letters indicate the different treatments.

Canonical correspondence analysis (CCA) revealed a positive correlation between *Lactobacillus* abundance and growth-related parameters, including WG, SGR, CF, FI, FCR, and FE (permutation test, *p* = 0.043; Fig. 5).

### Functional metagenomics prediction

PICRUSt2 analysis predicted 430 functional pathways, with biosynthesis pathways enriched in W and L+I treatments, as well as in some control samples clustering with W. The most abundant predicted functional categories were amino acid metabolism, carbohydrate metabolism, membrane transport, energy metabolism, cofactors and vitamins, translation, and nucleotide metabolism (Fig. S4).

## Discussion

Farmed *C. estor* continues to exhibit suboptimal performance, a trend linked to limitations in formulated diets and reduced intestinal microbiota diversity (Amillano-Cisneros et al., 2022). In this study, supplemented diets increased final body length and weight, weight gain, specific growth rate, condition factor, and feed efficiency, while reducing feed conversion ratio (Table 1). These findings provide species-specific evidence supporting the use of functional dietary additives in native freshwater fish and are consistent with reports from other aquaculture species in which synbiotics enhanced growth and nutrient utilization (Thakur et al., 2025; Wisastra et al., 2025).

The observed improvements in performance likely reflect multiple complementary mechanisms associated with probiotic and synbiotic supplementation. Probiotics can enhance digestive efficiency through the production of extracellular enzymes that facilitate nutrient breakdown and absorption, as well as by inducing structural modifications in the intestinal epithelium that increase absorptive surface area. In addition, probiotic bacteria produce metabolites and antimicrobial compounds that suppress opportunistic pathogens, thereby improving gut health and nutrient allocation. In the present study, increased abundance of lactic acid bacteria (LAB), particularly in synbiotic treatments (Fig. 1), was positively associated with growth performance (Fig. 5), suggesting that these mechanisms may operate in *C. estor*. Certain LAB are also capable of synthesizing essential amino acids, including isoleucine, leucine, lysine, and valine, which may further contribute to enhancing growth and feed efficiency (Dai, Wu & Zhu, 2011; Neis, Dejong & Rensen, 2015; Lim et al., 2019).

Although prebiotic supplementation has been shown to alter muscle fatty acid profiles in some fish species, no significant changes were observed in *C. estor* in the present study (Table S2). This apparent stability likely reflects species-specific lipid metabolism characterized by a strong endogenous capacity to produce and maintain high DHA levels regardless of dietary modulation (Fonseca-Madrigal et al., 2014; Martínez-Palacios et al., 2020). From a nutritional perspective, this consistency may represent an advantage, ensuring stable fillet quality while allowing performance gains through microbiota-targeted dietary strategies. Longer feeding trials or alternative prebiotic formulations may be required to determine whether lipid metabolism can be modulated under different conditions.

A limitation of this study was the absence of probiotic strains isolated directly from the intestinal microbiota of *C. estor*. Host-derived probiotics may enhance colonization potential and functional compatibility due to host–microbe specificity within gut ecosystems. Another limitation was the reduced tank-level replication for one dietary treatment, which constrained inference at the tank level. In addition, dissolved oxygen levels averaged approximately four mg/L, which is below the optimal range for this fish species (Vigueras-Velázquez et al., 2020). Despite this, fish maintained normal feeding behavior and showed no apparent signs of physiological stress throughout the experiment, possibly reflecting insufficient aeration within the system. This factor should be considered in the design of future experiments. However, analysis of a large number of individually sampled fish using mixed-effects models allowed robust detection of consistent dietary associations. Consequently, results are interpreted conservatively and provide a foundation for future fully replicated trials.

Alpha diversity indices differed significantly only between treatments L and W relative to L+I (Fig. 2), and overall supplemented groups tended to exhibit higher microbial richness than controls. Such patterns have previously been associated with enhanced host resilience and metabolic gut stability (Aryati et al., 2021; Fachri et al., 2024). Increased gut microbial diversity is widely regarded as an indicator of a more stable intestinal ecosystem, conferring resistance to pathogen invasion and environmental stressors (Larsen & Claassen, 2018). Therefore, even modest increases in microbial richness may contribute to improved intestinal stability in cultured *C. estor*.

Beta diversity and muscle fatty acid profiles did not show significant differences among treatments (Fig. 2; Table S5). However, LDA analysis identified *Kosmotoga, Limosilactobacillus, Sphingomonas,* and *Vibrio* as differentially abundant in fish receiving the yeast cell wall prebiotic (Fig. 3). These taxa are commonly associated with carbohydrate fermentation, nutrient cycling, and microbial interactions that may enhance gut functionality. Several members of *Limosilactobacillus, Sphingomonas,* and *Vibrio* have previously been described as beneficial probiotics in aquaculture systems, supporting their potential functional relevance in *C. estor* (Choudhary & Mahadevan, 2020; Thakur et al., 2025).

Core microbiota analysis further revealed nine genera (*Bacillus, Citrobacter, Cutibacterium, Lactobacillus, Pseudomonas, Spiroplasma, Stenotrophomonas, Streptococcus,* and *Thermogemmatispora*) (Fig. 4). Among these, lactic acid bacteria such as *Lactobacillus* and *Streptococcus* are consistently reported as core or recurrent members of the gut microbiota in both freshwater fish and marine species, where they are associated with digestive processes and immune modulation (Merrifield & Carnevali, 2014; Ringø  et al., 2018). Other genera detected here, including *Bacillus* and *Pseudomonas*, have likewise been widely reported across diverse freshwater and marine fish hosts, supporting the existence of a broadly conserved fish gut microbiota core with potential physiological and nutritional relevance (Egerton et al., 2018; Yajima et al., 2023). Collectively, these taxonomic patterns suggest that dietary supplementation selectively promotes bacterial groups potentially involved in nutrient metabolism and host health.

PICRUSt2-based functional prediction indicated that dietary treatments influenced the predicted abundance of microbial pathways related to amino acid, carbohydrate, energy, cofactor, vitamin, and nucleotide metabolism (Fig. S4). However, these functional predictions should be interpreted cautiously because they are inferred from 16S rRNA gene data rather than directly measured microbial gene expression. Despite this limitation, the enrichment of biosynthetic pathways in supplemented treatments aligns with the observed improvements in growth and feed efficiency, suggesting that microbiota-mediated metabolic contributions may support host performance. Similar diet-associated shifts in microbial functional potential have been reported in other fish species. In Atlantic salmon, dietary lipid composition altered predicted gut microbial pathways related to energy and lipid metabolism (Huyben et al., 2020), while functional profiling of the rainbow trout intestinal microbiome revealed enrichment of pathways involved in carbohydrate metabolism and nutrient transport under different rearing and dietary conditions (Lyons et al., 2017). Together, these studies support the interpretation that diet-driven modulation of the gut microbiota can influence microbial metabolic potential in fish, consistent with the patterns observed in *C. estor*.

## Conclusions

Dietary supplementation with synbiotics and a yeast cell wall prebiotic improved growth performance in *Chirostoma estor*, increasing weight gain, specific growth rate, and feed efficiency while maintaining stable muscle fatty acid profiles, potentially in association with microbiota-related nutritional effects. Although differences in alpha diversity were limited, supplemented treatments showed higher microbial richness and increased abundance of lactic acid bacteria, particularly *Lactobacillus* spp. Taxonomic analyses identified differentially abundant genera such as *Kosmotoga, Limosilactobacillus, Sphingomonas,* and *Vibrio*, indicating that dietary supplementation selectively influences bacterial groups potentially involved in nutrient metabolism and gut functionality. Functional predictions suggested an enrichment of microbial pathways associated with amino acid, carbohydrate, energy, cofactor, vitamin, and nucleotide metabolism in supplemented groups. These predicted metabolic shifts align with the observed improvements in growth and feed efficiency, supporting the hypothesis that microbiota-mediated metabolic processes contribute to host nutritional performance. The recurrent detection of specific bacterial genera within the core microbiota also indicates that the intestinal microbiome of *C. estor* may represent a source of host-associated bacteria for future probiotic and functional evaluation. Overall, these findings indicated that synbiotic and yeast cell wall supplementation were associated with improved growth performance and gut microbiota modulation in *C*. *estor*. In a broader context, this study contributes to ongoing efforts to develop synbiotic-based strategies in fish nutrition and highlights the importance of microbiome-informed approaches that may support more sustainable aquaculture practices, particularly for native freshwater fish species with culture potential.

##  Supplemental Information

10.7717/peerj.21435/supp-1 Supplemental Information 1 Supplementary figures and tables

10.7717/peerj.21435/supp-2 Supplemental Information 2 Dataset 1, Fig. 1 Raw data for Fig. 1

10.7717/peerj.21435/supp-3 Supplemental Information 3 Dataset 2, Table 2 Raw data for Table 2

10.7717/peerj.21435/supp-4 Supplemental Information 4 Dataset, Table 3 Raw data for Table 3

10.7717/peerj.21435/supp-5 Supplemental Information 5 ARRIVE checklist

## Additional Information and Declarations

### DNA Deposition

The following information was supplied regarding the deposition of DNA sequences: Sequences are available under BioProject ID PRJNA796804 in SRA-NCBI: from SAMN25047428 to SAMN25047469.

### Data Availability

The following information was supplied regarding data availability: The raw data is available in the Supplemental Files. Sequences are available under BioProject ID PRJNA796804 in SRA-NCBI: from SAMN25047428 to SAMN25047469.
