# Supplementary material for: Dietary prebiotics and synbiotics modulate gut microbiota and improve growth performance of Mexican pike silverside Chirostoma estor
Source: PeerJ. 2026 Jul 1;14:e21435. doi: 10.7717/peerj.21435 (PMC13332718; doi:10.7717/peerj.21435)
Supplement: Supplemental Information 1 [file peerj-14-21435-s001.docx]

### **Supplementary tables and figures - Amillano et al. 2026**

**A)**

**
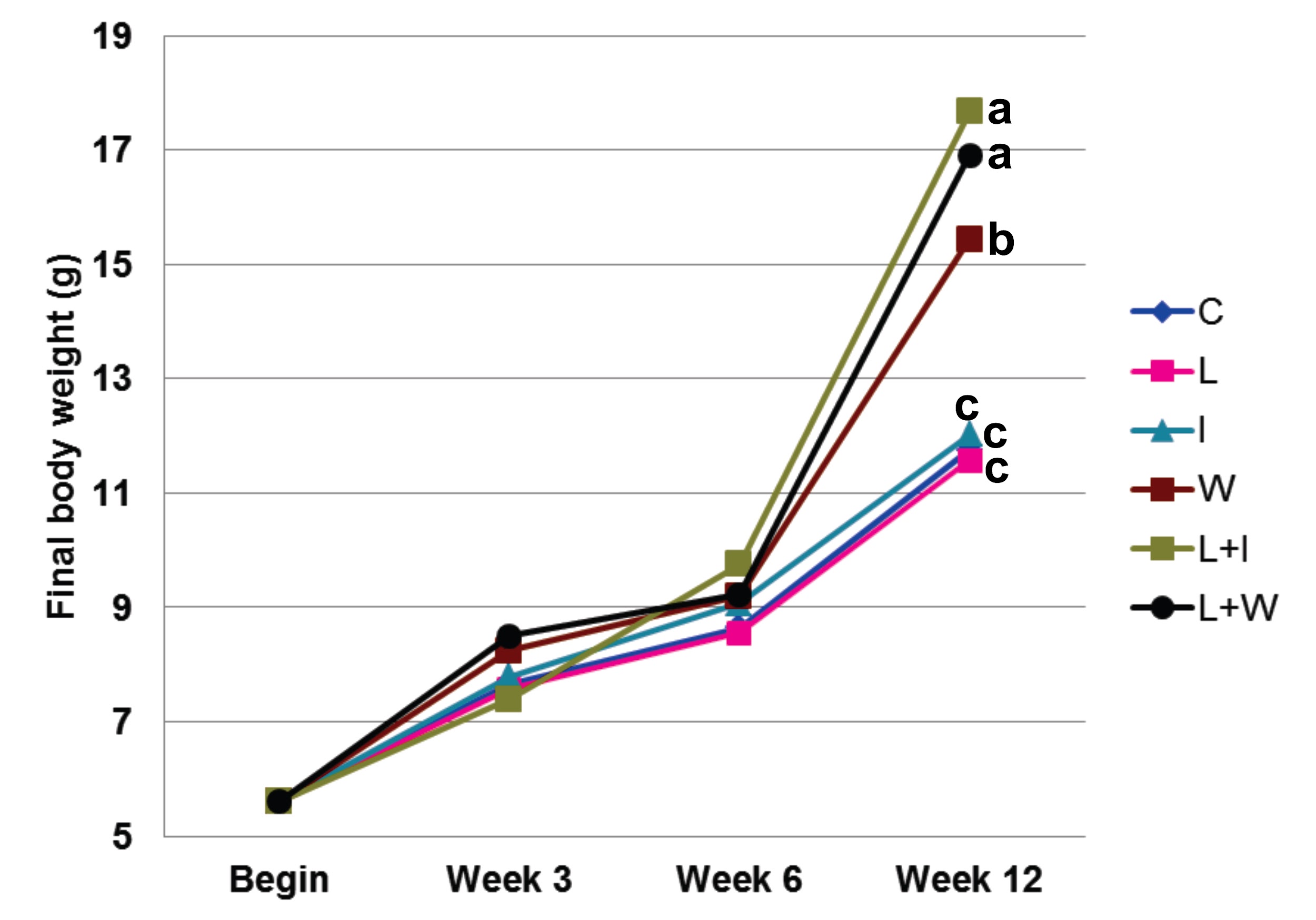
**

**B)**

**
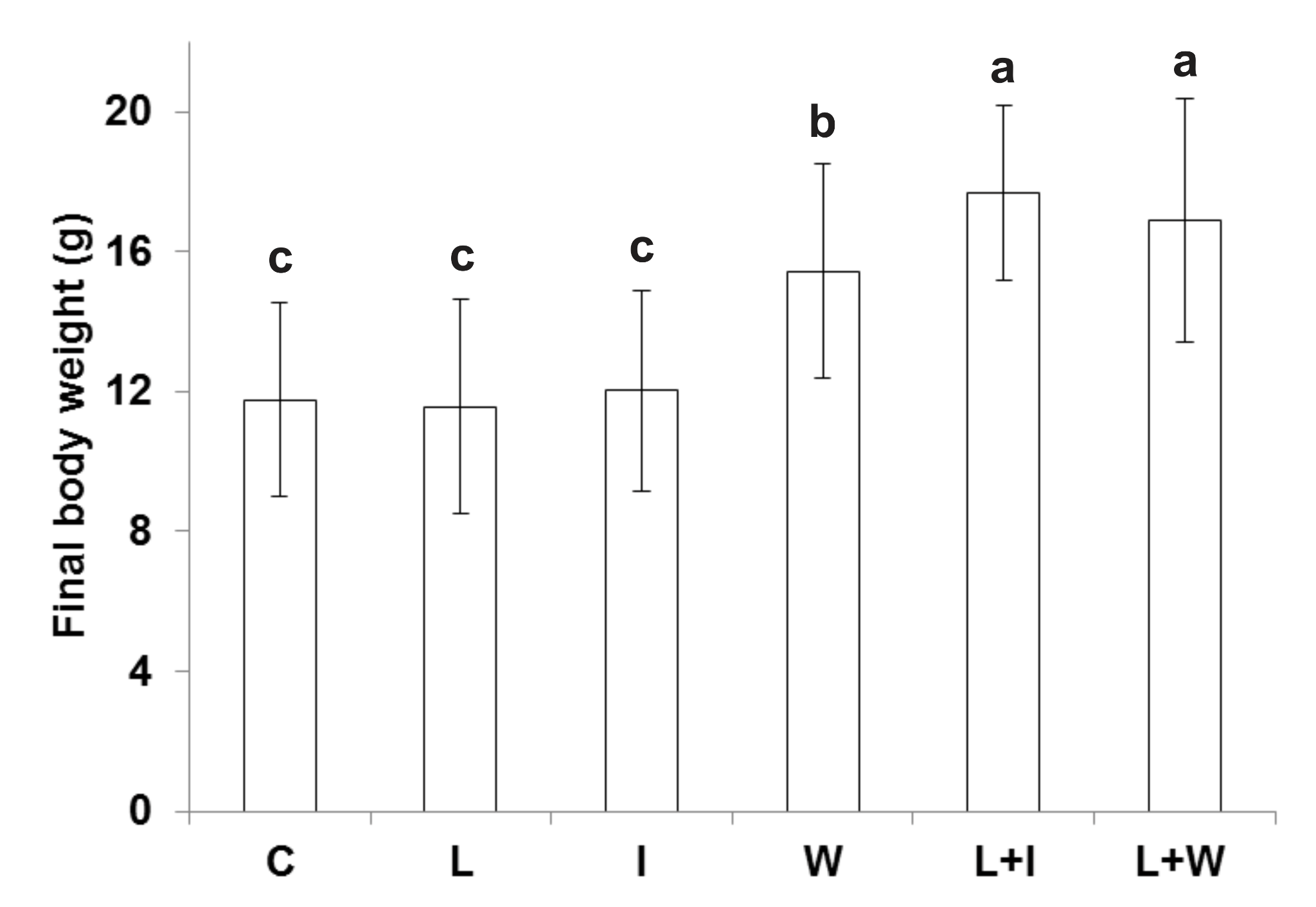
**

**Figure S1. Growth performance of pike silverside (*Chirostoma estor*)**. A) Dynamic growth recorded at weeks 3, 6, and 12, with 20 fish measured per sampling point. B) Final body weight (g) after 12 weeks of feeding experimental diets: Control (C), Lactobacillus (L), Inulin (I), Yeast cell wall (W), L. acidophilus + Inulin (L+I), and L. acidophilus + Cell wall (L+W).

**Figure S2. Differential phyla in the Control (C), *Lactobacillus* (L), Inulin, and Cell Wall treatments.** Linear Discriminant Analysis (LDA) of bacterial phyla inferred by LEfSe for phyla in the gut community within the different treatments. Differentiating feature analysis utilized the Kruskal-Wallis test with a raw p-value cutoff of 0.05. When no differences present no bars are shown.


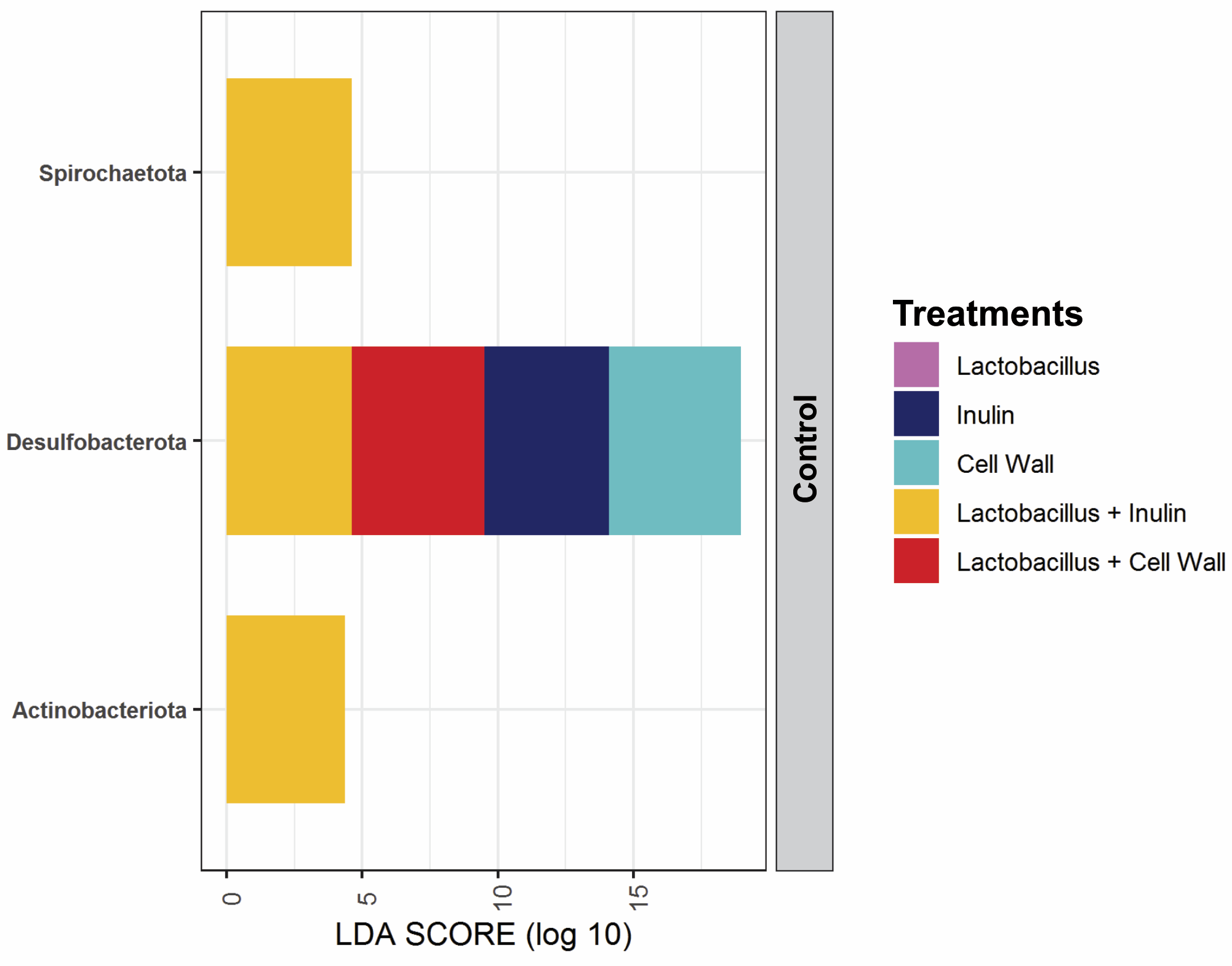


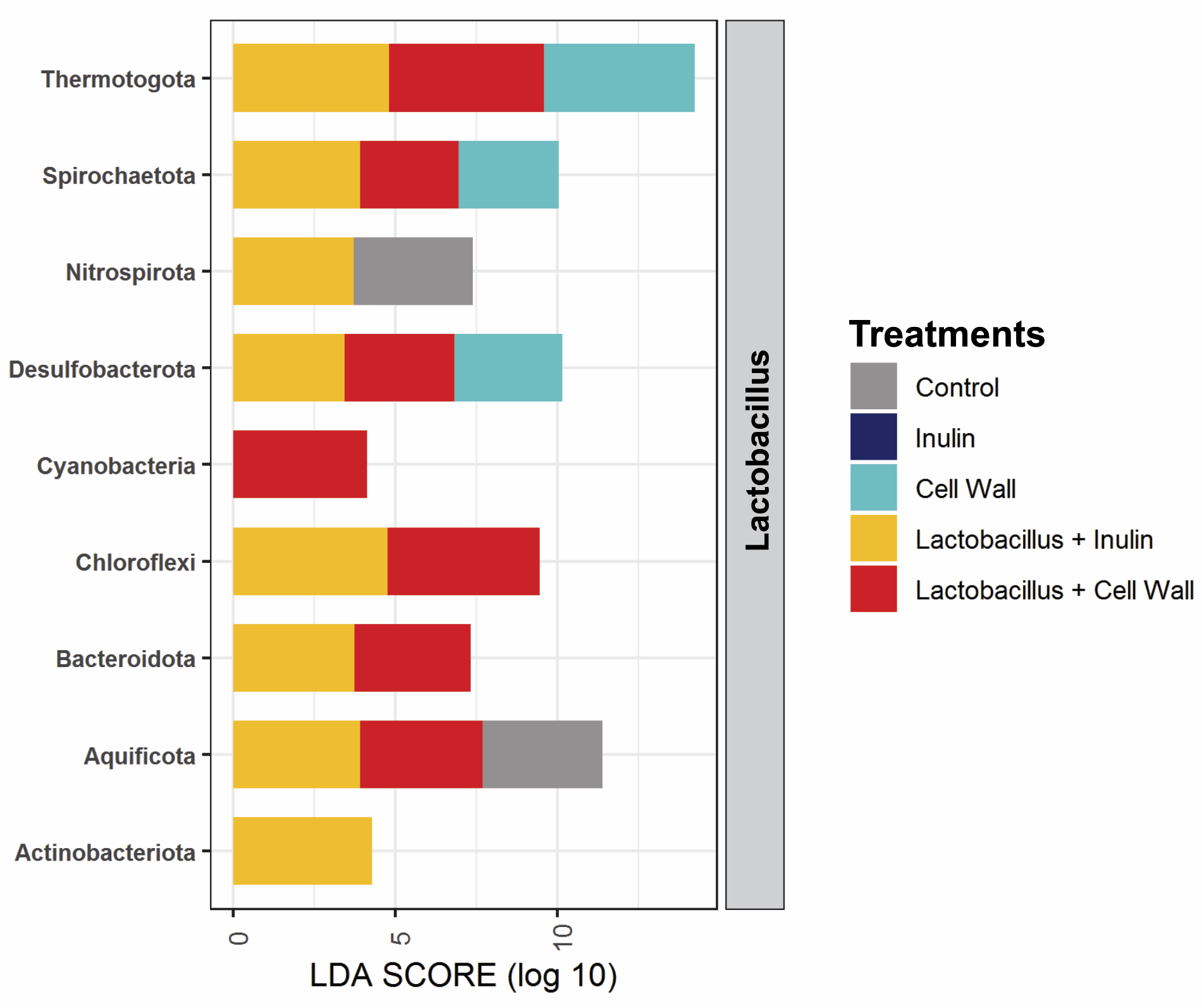


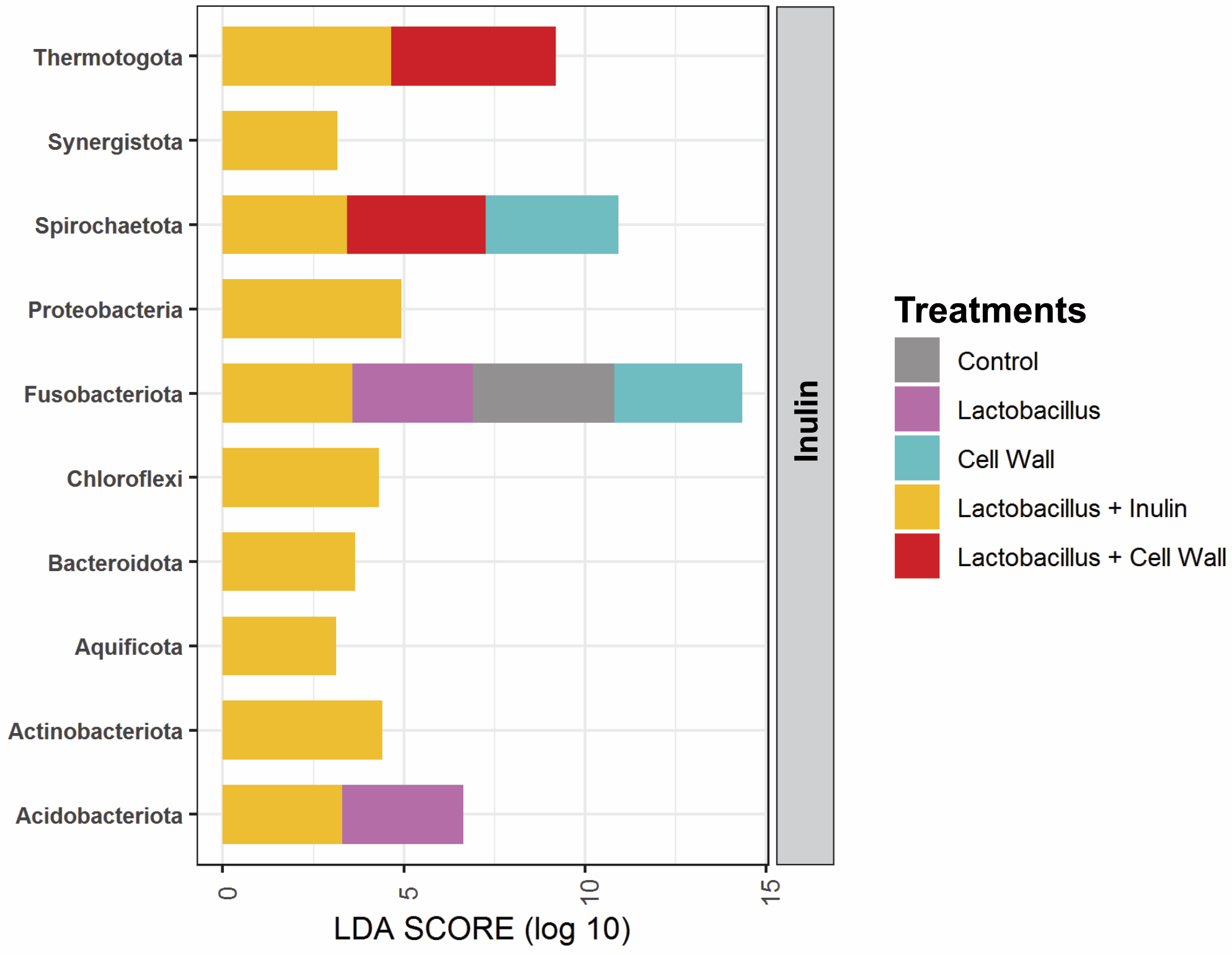


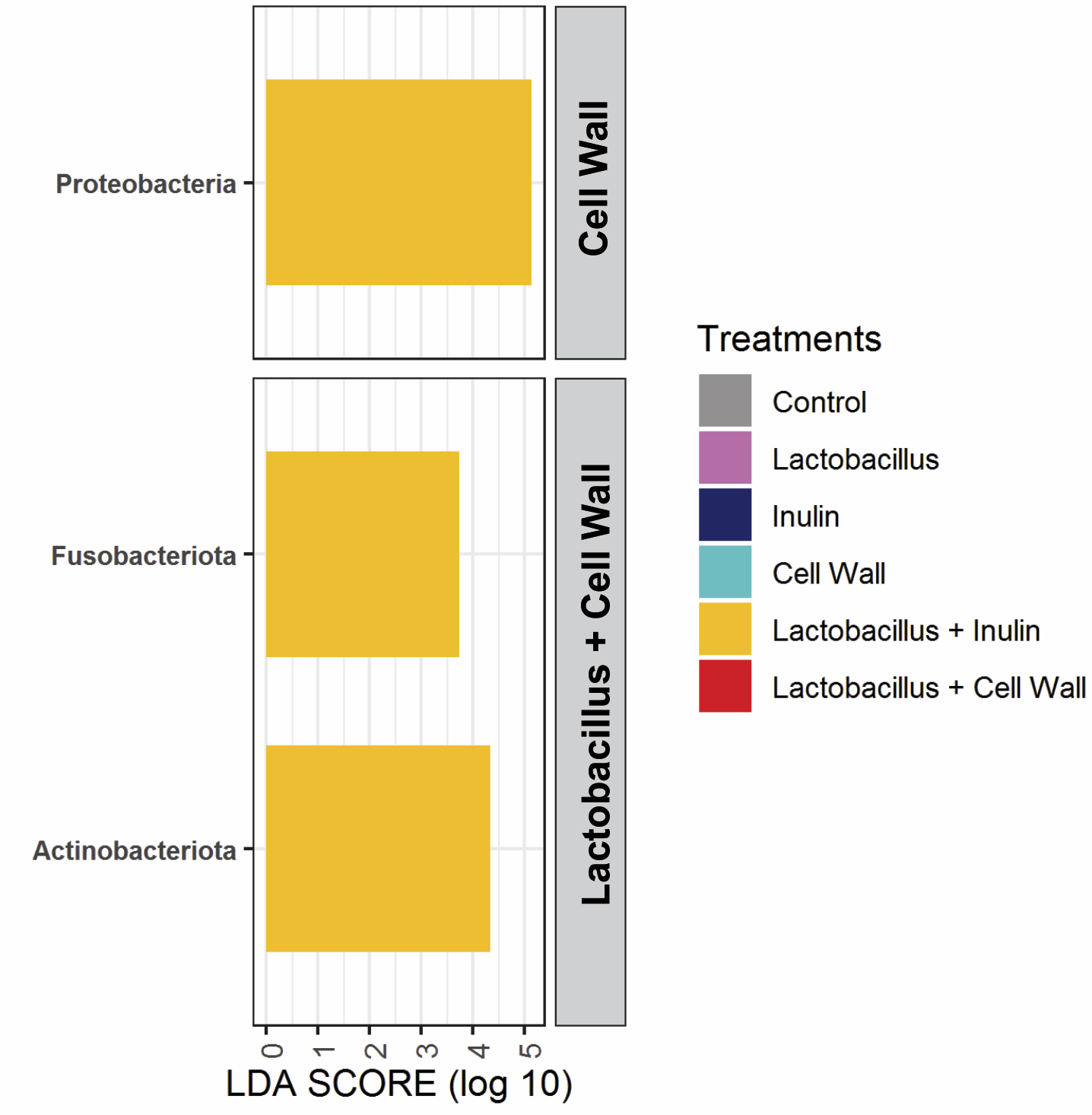


**Figure S3. Differential genera in the Control, *Lactobacillus* and Inulin treatments.** Linear Discriminant Analysis (LDA) of bacterial genera as inferred by LEfSe analysis for the gut community under Control (C), *L. acidophilus* (L), and Inulin (I) treatments. Differentiating feature analysis was conducted using the Kruskal-Wallis test with a raw p-value cutoff of 0.05.


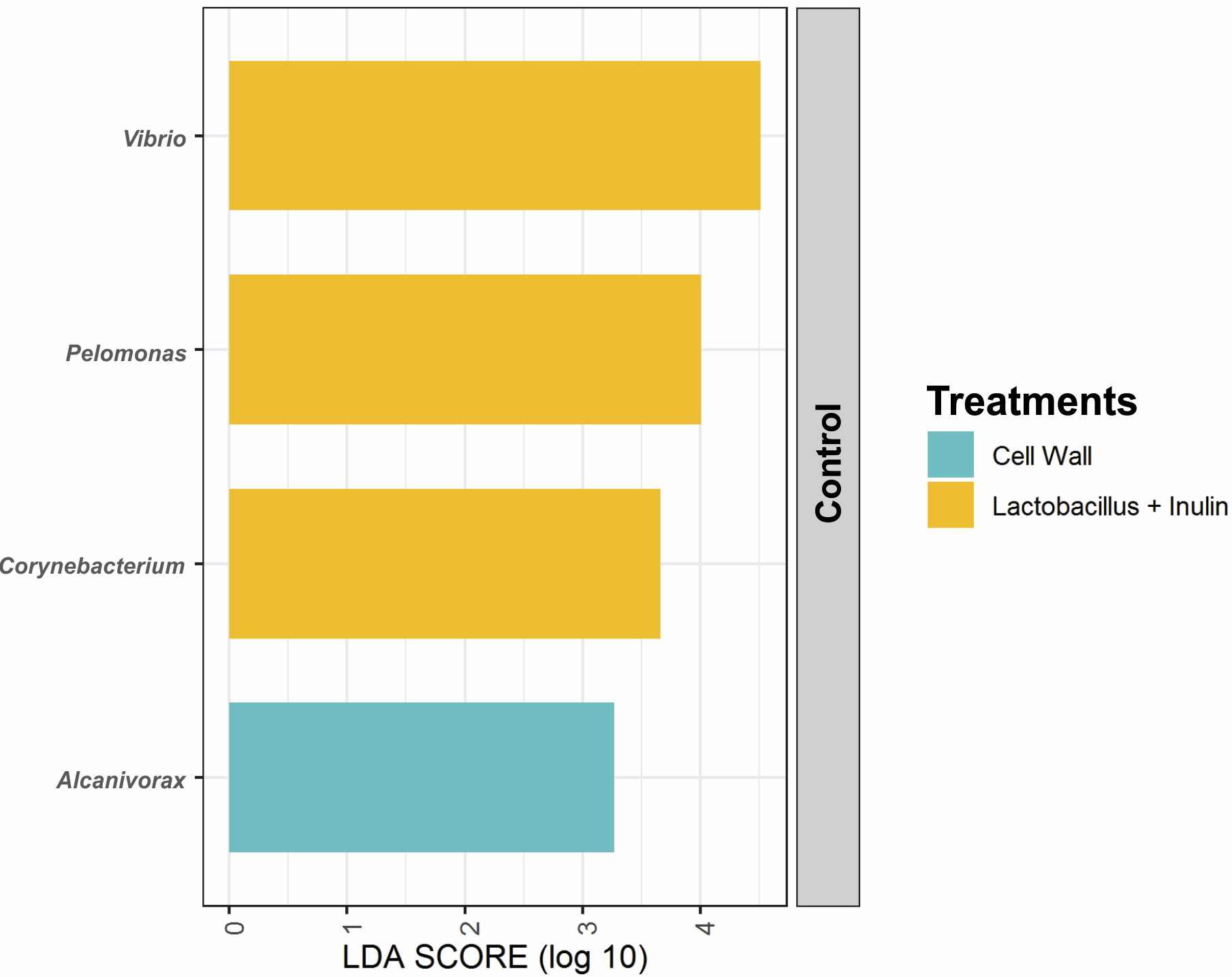


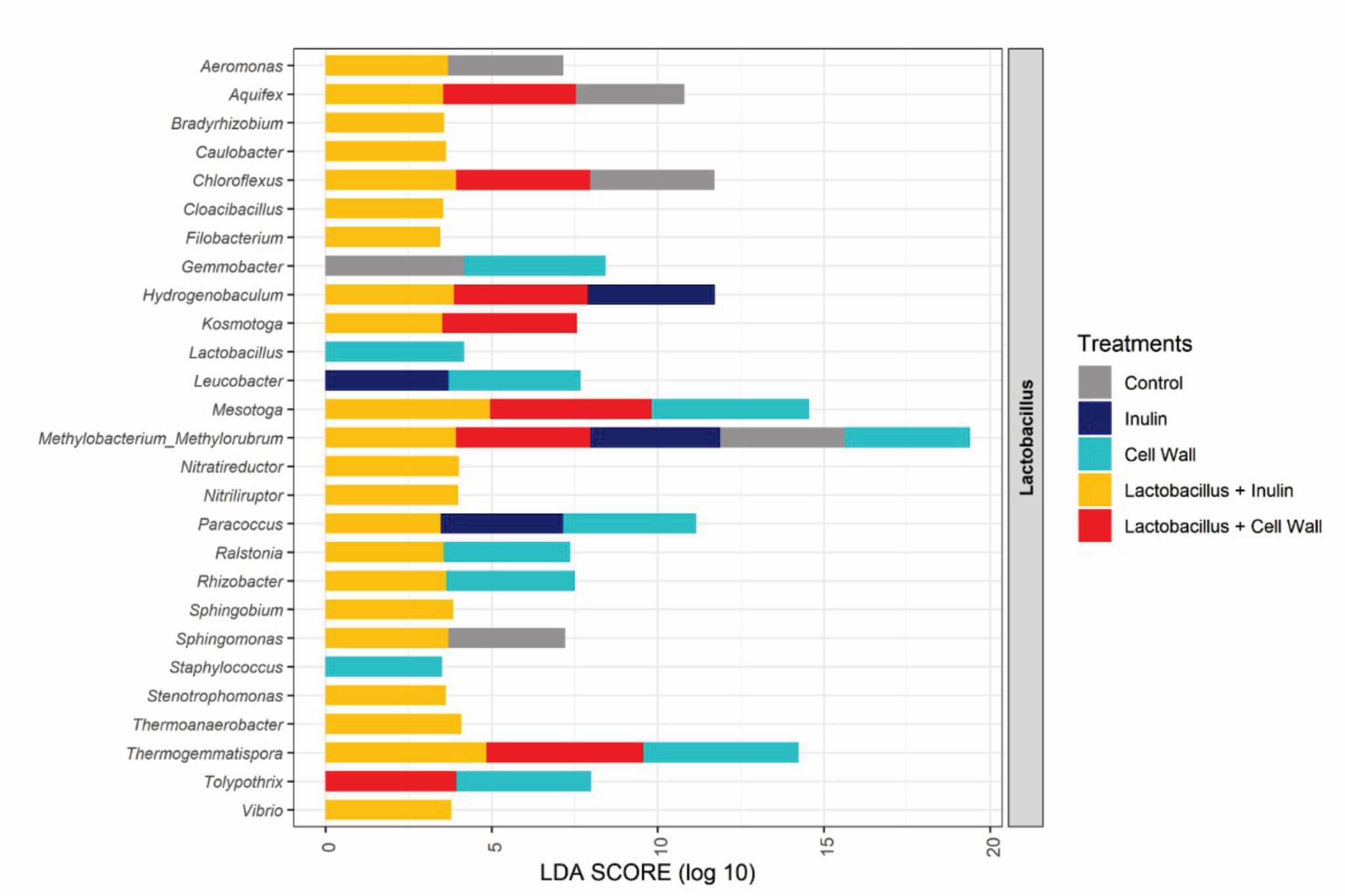


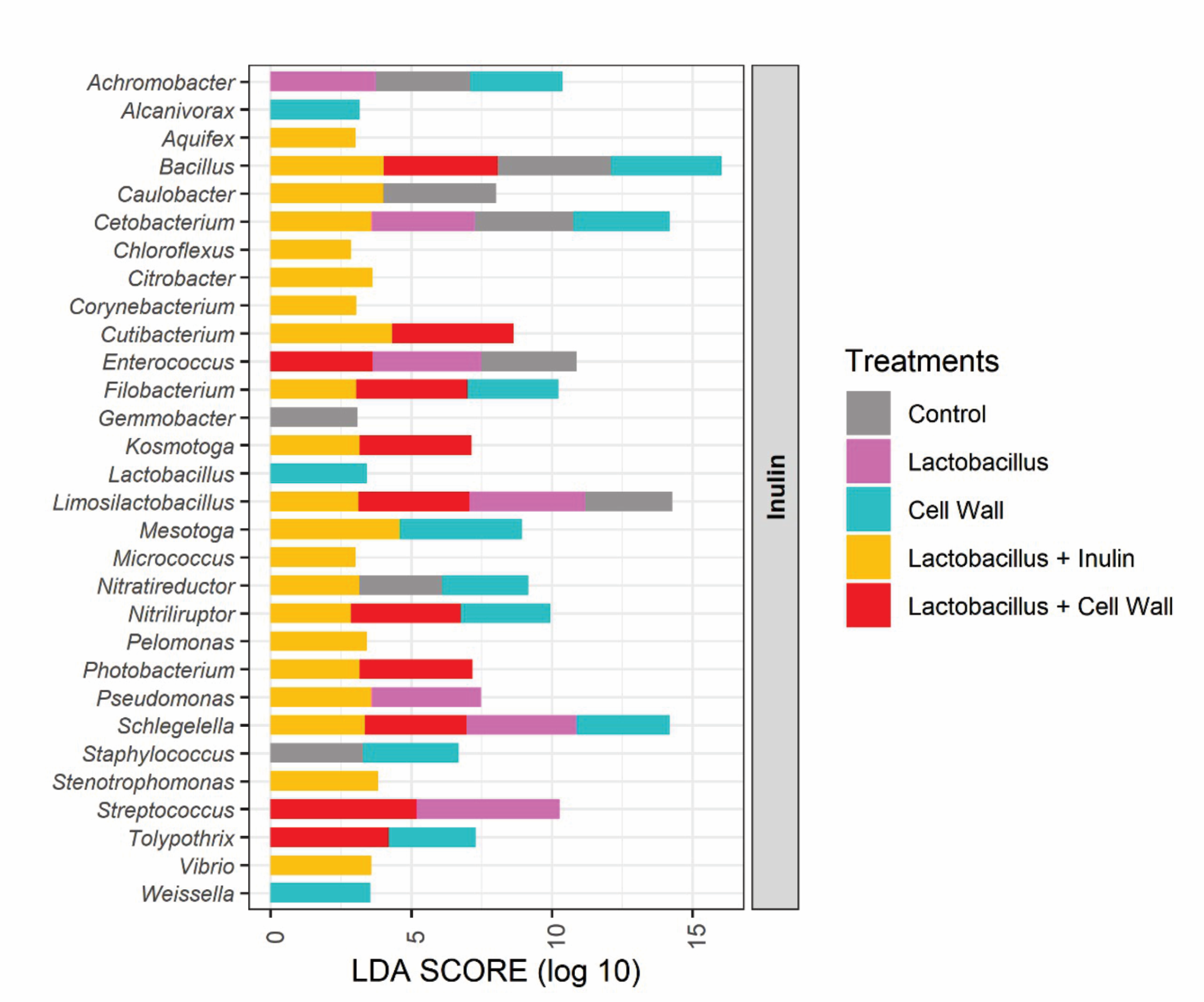


**Figure S4. Predicted metabolic pathways of the gut microbiota across treatments.** Heatmap and dendrogram displaying the 60 most abundant and differentially represented metabolic pathways. Higher relative abundance is indicated in red, and lower abundance in blue. Treatment groups are identified by the top color bars: Control (C, gray); *L. acidophilus* (L, magenta); Inulin (I, blue); Cell wall (W, cyan); *L. acidophilus* + Inulin (L+I, yellow); and *L. acidophilus* + Cell wall (L+W, red).


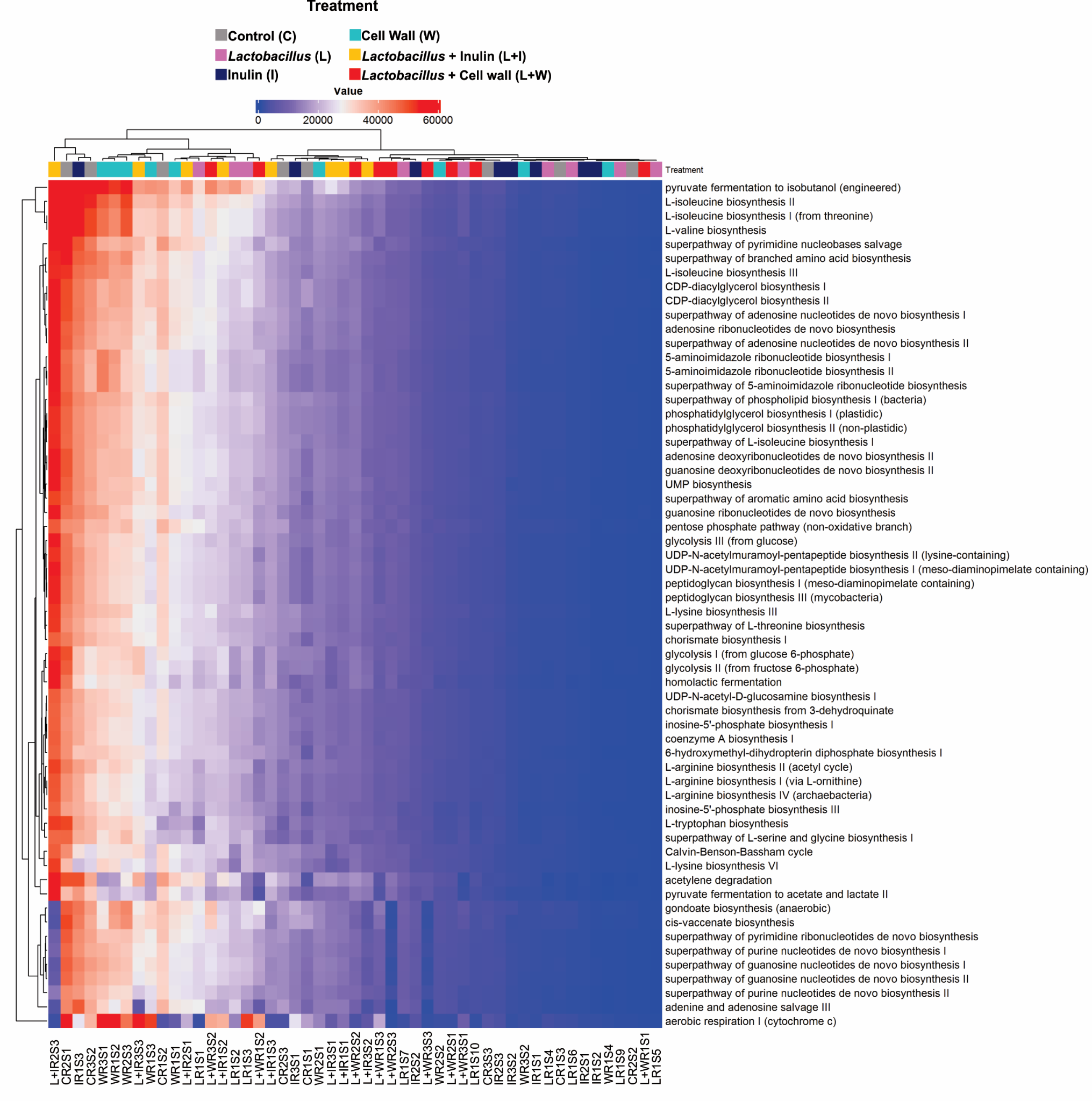


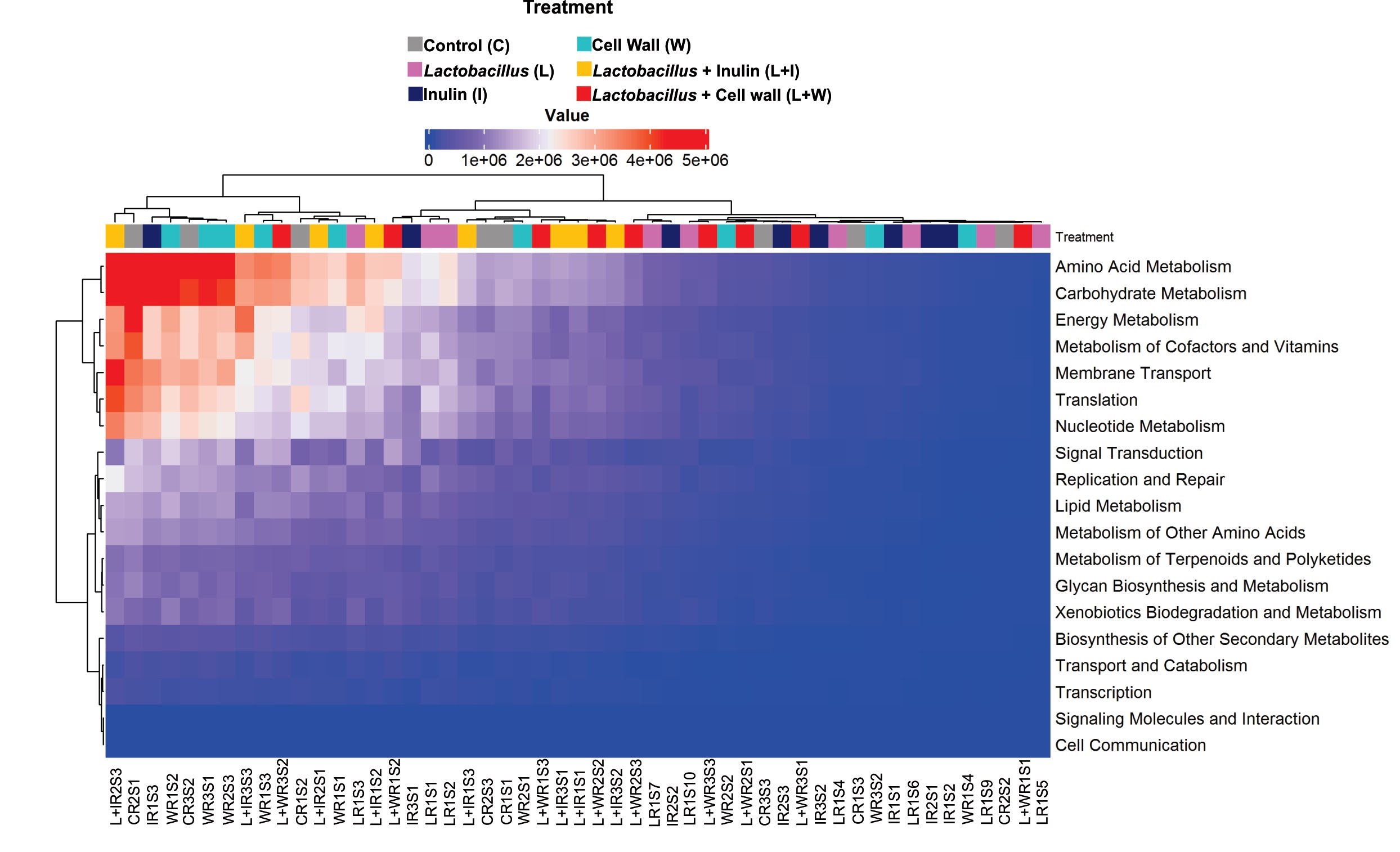


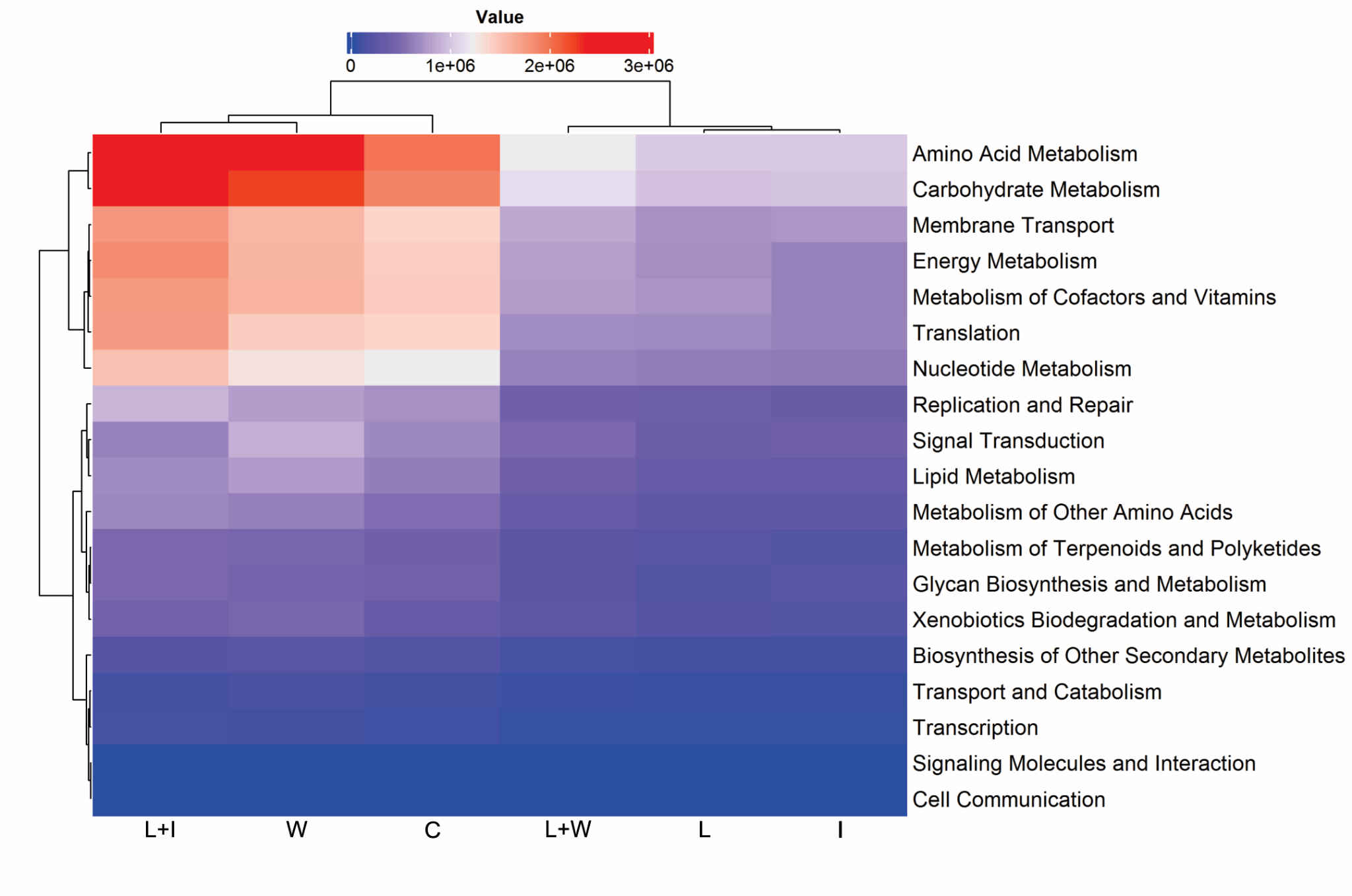


**Table S1.** Proximate composition of control, probiotic, prebiotics and synbiotics treatment diets.

|  |  | |  |  |  |  | |  |
| --- | --- | --- | --- | --- | --- | --- | --- | --- |
| Ingredient (g Kg^-1^) | | Treatments | | | | | | |
|  |  | **C** | **L** | **I** | **W** | **L+I** | **L+W** | |
| Marine protein sources^1^ | | 423.11 | 423.11 | 423.11 | 423.11 | 423.11 | | 423.11 |
| Soluble protein sources^2^ | | 199.20 | 199.20 | 199.20 | 199.20 | 199.20 | | 199.20 |
| Soy protein isolate | | 57.97 | 57.97 | 57.97 | 57.97 | 57.97 | | 57.97 |
| Canola oil | | 3.89 | 3.89 | 3.89 | 3.89 | 3.89 | | 3.89 |
| Corn starch | | 251.00 | 246.00 | 231.00 | 250.75 | 226.00 | | 245.75 |
| BHT (antioxidant) | | 0.50 | 0.50 | 0.50 | 0.50 | 0.50 | | 0.50 |
| Soy Lecitin | | 19.30 | 19.30 | 19.30 | 19.30 | 19.30 | | 19.30 |
| Vitamin premix* | | 15.00 | 15.00 | 15.00 | 15.00 | 15.00 | | 15.00 |
| Arabic gum | | 18.00 | 18.00 | 18.00 | 18.00 | 18.00 | | 18.00 |
| Sodium alginate | | 2.00 | 2.0 | 2.00 | 2.00 | 2.00 | | 2.00 |
| Rovimix Stay C-35* | | 3.40 | 3.40 | 3.40 | 3.40 | 3.40 | | 3.40 |
| Crystalline Taurine | | 10.00 | 10.00 | 10.00 | 10.00 | 10.00 | | 10.00 |
| Choline Chloride* | | 3.00 | 3.00 | 3.00 | 3.00 | 3.00 | | 3.00 |
| Mineral premix (Trace elements)* | | 15.00 | 15.00 | 15.00 | 15.00 | 15.00 | | 15.00 |
| Mineral premix (macro elements)* | | 10.00 | 10.00 | 10.00 | 10.00 | 10.00 | | 10.00 |
| Inulin | | 0.00 | 0.00 | 20.00 | 0.00 | 20.00 | | 0.00 |
| *Saccharomyces cerevisiae* cell wall | | 0.00 | 0.00 | 0.00 | 0.25 | 0.00 | | 0.25 |
| *Lactobacillus acidophilus*^3^ | | 0.00 | 5.00 | 0.00 | 0.00 | 5.00 | | 5.00 |
| Proximate composition (% ± SD) | |  |  |  |  |  | |  |
| Moisture | | 8.58 ± 0.01 | 8.90 ± 0.03 | 4.94 ± 0.17 | 7.50 ± 0.17 | 8.64 ± 0.09 | | 11.24 ± 0.14 |
| Ash | | 12.45 ± 0.17 | 14.72 ± 0.06 | 14.26 ± 0.15 | 12.47 ± 0.09 | 14.58 ± 0.16 | | 14.00 ± 0.33 |
| Lipid | | 6.53 ± 0.03 | 6.25 ± 0.07 | 6.23 ± 0.24 | 6.15 ± 0.18 | 5.88 ± 0.15 | | 4.65 ± 0.26 |
| Protein | | 41.03 ± 0.23 | 40.38 ± 0.18 | 42.46 ± 0.07 | 41.93 ± 0.07 | 40.11 ± 0.43 | | 39.02 ± 0.05 |

^1^Marine protein sources: Brown Fish Meal (Sardine), Krill Meal. ^2^Soluble protein sources: Egg albumin, Milk Whey, Calcium caseinate. ^3^Final concentration of *Lactobacillus* in *Lactobacillus*, *Lactobacillus* + inulin and *Lactobacillus* + cell wall treatments was 1x10^6^ CFU g/kg. *DSM Nutritional products. Ash, lipid and protein proximate composition is expressed as % wet weight. C, Control; L, Lactobacillus; I, inulin; W yeast cell wall; L+I, *L*. *acidophilus* + inulin; W, *L*. *acidophilus* + cell wall.

**Table S2.** **Fatty acid profile of eviscerated pike silverside (*Chirostoma estor*).** Mean fatty acid composition (± SD) of fish fed experimental diets for 12 weeks.

|  | **Treatments** | | | | | | |
| --- | --- | --- | --- | --- | --- | --- | --- |
| **Fatty acid** | **C** | **L** | **I** | **W** | **L+I** | **L+W** | **P- value** |
| C14:0 | 4.18 ± 0.26 | 3.45 ± 0.36 | 3.86 ± 0.20 | 4.15 ± 0.39 | 3.97 ± 0.62 | 3.66 ± 0.13 | 0.177 |
| C15:0 | 0.41± 0.01 | 0.38± 0.05 | 0.42 ± 0.04 | 0.45 ± 0.01 | 0.47 ± 0.06 | 0.45 ± 0.01 | 0.118 |
| C16:0 | 23.77 ± 2.73 | 23.35 ± 1.06 | 23.37 ± 0.64 | 24.06 ± 0.55 | 23.70 ± 1.18 | 23.83 ± 0.81 | 0.985 |
| C18:0 | 5.76 ± 1.32 | 6.16 ± 0.31 | 6.02 ± 0.51 | 5.35 ± 0.54 | 5.90 ± 1.03 | 5.83 ± 0.22 | 0.847 |
| Total SFA | 34.12 ± 3.80 | 33.33 ± 1.41 | 33.67 ± 1.10 | 34.01 ± 0.62 | 34.04 ± 1.54 | 33.78 ± 1.10 | 0.995 |
| C16:1n-7 | 5.05 ± 0.63 | 4.29 ± 0.43 | 4.60 ± 0.16 | 4.81 ± 0.46 | 4.43 ± 0.53 | 4.32 ± 0.21 | 0.28 |
| C18:1(n-7) | 3.93 ± 0.18 | 3.71 ± 0.29 | 3.70 ± 0.05 | 3.82 ± 0.16 | 3.70 ± 0.14 | 3.72 ± 0.12 | 0.515 |
| C18:1(n-9) | 18.67 ± 1.15 | 16.50 ± 2.04 | 16.87 ± 0.87 | 18.84 ± 1.05 | 15.96 ± 0.50 | 16.57 ± 1.08 | 0.0536 |
| C20:1(n-9, n-11) | 0.46 ± 0.03 | 0.39 ± 0.06 | 0.43 ± 0.06 | 0.50 ± 0.04 | 0.40 ± 0.08 | 0.45 ± 0.02 | 0.183 |
| Total MUFA | 28.11 ± 0.77 | 24.88 ± 2.81 | 25.60 ± 0.87 | 27.97 ± 1.49 | 24.28 ± 0.67 | 25.07 ± 1.18 | 0.0356 |
| C18:2(n-6) | 11.29 ± 1.47 | 10.59 ± 0.60 | 11.21 ± 0.28 | 11.27 ± 0.89 | 11.13 ± 0.77 | 10.96 ± 0.53 | 0.905 |
| C18:3(n-3) | 1.28 ± 0.35 | 1.16 ± 0.07 | 1.41 ± 0.05 | 1.41 ± 0.19 | 1.39 ± 0.24 | 1.41 ± 0.07 | 0.559 |
| C18:4(n-3) | 0.37 ± 0.16 | 0.31 ± 0.01 | 0.45 ± 0.07 | 0.43 ± 0.07 | 0.47 ± 0.13 | 0.45 ± 0.02 | 0.363 |
| C20:4(n-3) | 0.23 ± 0.07 | 0.23 ± 0.01 | 0.28 ± 0.01 | 0.24 ± 0.03 | 0.27 ± 0.03 | 0.26 ± 0.04 | 0.544 |
| C20:4(n-6)ARA | 2.21 ± 0.03 | 2.73 ± 0.40 | 2.45 ± 0.06 | 2.26 ± 0.24 | 2.54 ± 0.21 | 2.52 ± 0.14 | 0.103 |
| C20:5(n-3)EPA | 2.68 ± 0.54 | 3.00 ± 0.43 | 3.22 ± 0.35 | 3.55 ± 0.33 | 3.53 ± 0.55 | 3.13 ± 0.08 | 0.163 |
| C22:5(n-3) | 2.62 ± 0.53 | 2.98 ± 0.40 | 2.76 ± 0.30 | 2.37 ± 0.02 | 2.64 ± 0.08 | 2.67 ± 0.46 | 0.497 |
| C22:6(n-3)DHA | 17.09 ± 1.27 | 20.77 ± 3.52 | 18.95 ± 0.45 | 16.49 ± 1.78 | 19.51 ± 0.65 | 19.74 ± 1.64 | 0.102 |
| Total PUFA | 37.77 ± 4.33 | 41.79 ± 4.04 | 40.73 ± 0.70 | 38.02 ± 1.01 | 41.47 ± 0.95 | 41.15 ± 2.26 | 0.31 |
| ω3 | 24.27 ± 2.86 | 28.46 ± 4.23 | 27.07 ± 0.68 | 24.49 ± 1.66 | 27.80 ± 0.39 | 27.67 ± 1.95 | 0.198 |
| ω6 | 13.50 ± 1.48 | 13.33 ± 0.22 | 13.66 ± 0.22 | 13.53 ± 0.65 | 13.67 ± 0.57 | 13.48 ± 0.52 | 0.993 |
| PUFA/SFA | 1.13 ± 0.27 | 1.26 ± 0.17 | 1.21 ± 0.06 | 1.12 ± 0.02 | 1.22 ± 0.08 | 1.22 ± 0.11 | 0.791 |
| ω3/ω6 | 1.80 ± 0.03 | 2.14 ± 0.35 | 1.98 ± 0.06 | 1.82 ± 0.20 | 2.04 ± 0.06 | 2.05 ± 0.12 | 0.192 |
| DHA/EPA | 6.49 ± 0.79 **a** | 6.91 ± 0.36 **a** | 5.92 ± 0.56 **ab** | 4.65 ± 0.52 **b** | 5.63 ± 1.03 **ab** | 6.29 ± 0.36 **ab** | 0.0165 |

Different letters per row are shown where there is statistical significance between treatments (*p* < 0.05). C, Control; L, Lactobacillus; I, inulin; W yeast cell wall; L+I, *L*. *acidophilus* + inulin; W, *L*. *acidophilus* + cell wall. SFA, Saturated Fatty Acids; MUFA, Monounsaturated Fatty Acids; PUFA, Polyunsaturated Fatty Acids.

**Table S3. Relative abundance of dominant bacterial taxa.** Total mean relative abundance (%) ± SE (first column), followed by the mean relative abundance ± SE of the nine most prevalent phyla and two classes identified in the gut of pike silverside (*Chirostoma estor*) seed treated with probiotics, prebiotics, and synbiotics.

| **Phylum** | **Total abundance** | **Treatment** | | | | | |  |
| --- | --- | --- | --- | --- | --- | --- | --- | --- |
|  |  | **C** | **L** | **I** | **W** | **L+I** | **L+W** | **P value** |
|  | **(N=51)** | **(N=8)** | **(N=9)** | **(N=8)** | **(N=9)** | **(N=8)** | **(N=9)** |  |
| Firmicutes | 57.95 ± 4.48 | 50.66 ± 13.25 **a** | 43.76 ± 12.47 **a** | 57.47 ± 7.87 **a** | 57.78 ± 9.42 **a** | 76.16 ± 8.44 **a** | 63.04 ± 12.43 **a** | 0.5197 |
| Gammaproteobacteria | 11.77 ± 2.23 | 11.67 ± 4.45 **ab** | 8.20 ± 3.12 **ab** | 16.29 ± 7.07 **a** | 11.17 ± 3.75 **a** | 2.54 ± 1.03 **b** | 20.19 ± 8.69 **a** | 0.1483 |
| Thermotogota | 6.60 ± 1.64 | 11.56 ± 6.75 **ab** | 12.65 ± 4.00 **a** | 8.42 ± 3.53 **a** | 5.08 ± 4.24 **ab** | 0.36 ± 0.23 **b** | 1.61 ± 0.96 **b** | 0.03036 |
| Alphaproteobacteria | 5.56 ± 1.13 | 1.67 ± 0.70 **b** | 11.98 ± 4.35 **a** | 4.16 ± 1.02 **a** | 6.00 ± 1.91 **ab** | 1.00 ± 0.37 **b** | 7.46 ± 3.39 **ab** | 0.04246 |
| Chloroflexi | 4.94 ± 1.18 | 7.28 ± 4.22 **a** | 11.27 ± 3.60 **a** | 3.91 ± 1.76 **a** | 4.66 ± 3.12 **ab** | 0.34 ± 0.14 **b** | 1.83 ± 1.12 **ab** | 0.04968 |
| Actinobacteriota | 4.86 ± 1.16 | 4.48 ± 1.71 **a** | 4.09 ± 1.61 **a** | 5.88 ± 1.33 **a** | 9.85 ± 5.58 **a** | 0.44 ± 0.19 **b** | 4.03 ± 2.20 **a** | 0.01517 |
| Cyanobacteria | 4.67 ± 1.70 | 3.93 ± 3.29 **a** | 3.26 ± 1.62 **a** | 0.34 ± 0.09 **a** | 2.25 ± 1.74 **a** | 18.65 ± 8.86 **a** | 0.56 ± 0.21 **a** | 0.2583 |
| Desulfobacterota | 1.29 ± 0.81 | 7.23 ± 4.88 **a** | 0.45 ± 0.13 **ab** | 0.30 ± 0.18 **b** | 0.11 ± 0.08 **b** | 0.06 ± 0.02 **b** | 0.01 ± 0.01 **b** | 0.001286 |
| Bacteroidota | 0.66 ± 0.10 | 0.85 ± 0.29 **ab** | 0.98 ± 0.26 **a** | 1.02 ± 0.34 **a** | 0.68 ± 0.20 **ab** | 0.17 ± 0.14 **b** | 0.25 ± 0.09 **b** | 0.06763 |
| Fusobacteriota | 0.42 ± 0.11 | 0.17 ± 0.08 **bc** | 0.46 ± 0.35 **bc** | 0.81 ± 0.25 **a** | 0.55 ± 0.39 **bc** | 0.05 ± 0.03 **c** | 0.46 ± 0.17 **ab** | 0.04405 |
| Aquificota | 0.40 ± 0.12 | 0.18 ± 0.11 **ab** | 1.24 ± 0.40 **a** | 0.14 ± 0.05 **ab** | 0.55 ± 0.46 **a** | 0.02 ± 0.01 **b** | 0.18 ± 0.11 **ab** | 0.03967 |

C, Control; L, Lactobacillus; I, inulin; W yeast cell wall; L+I, L. acidophilus + inulin; W, L. acidophilus + cell wall. Different letters per row indicate statistical significance between treatments abundances (*p* < 0.05).

**Table S4.** **Alpha diversity analysis.** Richness (Chao1) and Shannon diversity (means ± SD) of gut microbiota communities across treatments: Control (C), *L. acidophilus* (L), Inulin (I), Cell wall (W), *L. acidophilus* + inulin (L+I), and *L. acidophilus* + cell wall (L+W). Different letters within the same column indicate statistically significant differences between treatments (Kruskal-Wallis or ANOVA, *p* < 0.05).

|  | **Alpha diversity index** | |
| --- | --- | --- |
| **Treatment** | Chao1 | Shannon |
| **C** | 1619.95 ± 854.29 **a** | 2.80 ± 0.40 **ab** |
| **L** | 3435.33 ± 1604.63 **a** | 3.36 ± 0.51 **a** |
| **I** | 2270.55 ± 708.75 **a** | 3.18 ± 0.53 **ab** |
| **W** | 2227.30 ± 1580.91 **a** | 3.38 ± 0.47 **a** |
| **L+I** | 1785.17 ± 784.33 **a** | 2.59 ± 0.49 **b** |
| **L+W** | 2545.80 ± 1153.38 **a** | 3.16 ± 0.43 **ab** |
| **P value** | 0.1652 | 0.01322 |

**Table S5.** **Beta diversity analysis.** Analysis of similarity (ANOSIM) and Permutational multivariate analysis of variance (Adonis) of gut microbiota communities in treatments.

| **Statistics** | **Bray-Curtis** |  |  |
| --- | --- | --- | --- |
| ANOSIM |  |  |  |
| Permutation N | 999 |  |  |
| R | 0.05908 |  |  |
| *p* (same) | 0.071 |  |  |
| Pairwise comparisons (adonis) | *p* value | R^2^ | F value |
| C vs L | 0.155 | 0.0936 | 1.5490 |
| C vs I | 0.371 | 0.0683 | 1.0260 |
| C vs W | 0.447 | 0.0546 | 0.8661 |
| C vs L+I | 0.237 | 0.0822 | 1.2534 |
| C vs L+W | 0.228 | 0.0776 | 1.2613 |
| L vs I | 0.213 | 0.0830 | 1.3573 |
| L vs W | **0.036** | 0.1515 | 2.8561 |
| L vs L+I | **0.003** | 0.2108 | 4.0077 |
| L vs L+W | 0.232 | 0.0764 | 1.3230 |
| I vs W | 0.102 | 0.1087 | 1.8292 |
| I vs L+I | **0.015** | 0.1655 | 2.7770 |
| I vs L+W | 0.167 | 0.0932 | 1.5410 |
| W vs L+I | **0.040** | 0.1314 | 2.2686 |
| W vs L+W | 0.100 | 0.1051 | 1.8799 |
| L+I vs L+W | 0.094 | 0.1034 | 1.7299 |

C, Control; L, *Lactobacillus acidophilus*; I, Inulin; W, Cell wall; L+I, *L*. *acidophilus* + Inulin; L+W, *L*. *acidophilus* + Cell wall. Significant *p* values in bold.

**Table S6. Pair LDA scores.** Genera that showed positive values and p<0.05 through treatment pair comparisons.

| **Genus** | **Pair comparison** | **Positive value** | **LDA score** | **p** |
| --- | --- | --- | --- | --- |
| Alcanivorax | C & W | C | 3.27 | 0.018 |
| Vibrio | C & L+I | C | 4.51 | 0.027 |
| Pelomonas | C & L+I | C | 4.01 | 0.046 |
| Corynebacterium | C & L+I | C | 3.66 | 0.039 |
| Kosmotoga | W & I | W | 3.00 | 0.046 |
| Limosilactobacillus | W & I | W | 3.13 | 0.029 |
| Sphingomonas | W & L+I | W | 3.75 | 0.040 |
| Vibrio | W & L+I | W | 3.77 | 0.032 |
| Achromobacter | L+W & C | L+W | 5.13 | 0.001 |
| Lactobacillus | L+W & C | L+W | 5.14 | 0.011 |
| Achromobacter | L+W & L | L+W | 4.02 | 0.009 |
| Citrobacter | L+W & L | L+W | 4.19 | 0.030 |
| Lactobacillus | L+W & I | L+W | 5.14 | 0.009 |
| Enhydrobacter | L+W & I | L+W | 3.57 | 0.033 |
| Achromobacter | L+W & W | L+W | 4.28 | 0.041 |
| Staphylococcus | L+W & W | L+W | 4.25 | 0.013 |
| Schlegelella | L+W & W | L+W | 4.14 | 0.023 |
| Lactobacillus | L+W & W | L+W | 5.24 | 0.005 |
| Caulobacter | L+W & L+I | L+W | 4.06 | 0.027 |
| Cetobacterium | L+W & L+I | L+W | 3.55 | 0.041 |
| Citrobacter | L+W & L+I | L+W | 4.40 | 0.007 |
| Enhydrobacter | L+W & L+I | L+W | 3.74 | 0.024 |
| Nitratireductor | L+W & L+I | L+W | 3.62 | 0.025 |
| Raoultella | L+W & L+I | L+W | 3.92 | 0.040 |
| Sphingomonas | L+W & L+I | L+W | 3.79 | 0.029 |
| Stenotrophomonas | L+W & L+I | L+W | 4.35 | 0.0005 |
| Vibrio | L+W & L+I | L+W | 3.98 | 0.029 |
| Achromobacter | L+I & C | L+I | 3.99 | 0.011 |
| Enterococcus | L+I & C | L+I | 3.92 | 0.027 |
| Lactobacillus | L+I & C | L+I | 5.12 | 0.016 |
| Lactococcus | L+I & C | L+I | 4.24 | 0.034 |
| Weissella | L+I & C | L+I | 4.17 | 0.034 |
| Enterococcus | L+I & L | L+I | 3.70 | 0.003 |
| Streptococcus | L+I & L | L+I | 4.95 | 0.034 |
| Lactobacillus | L+I & I | L+I | 5.08 | 0.006 |
| Alcanivorax | L+I & W | L+I | 4.14 | 0.002 |
| Lactobacillus | L+I & W | L+I | 5.12 | 0.0007 |
| Lactococcus | L+I & W | L+I | 4.49 | 0.025 |
| Staphylococcus | L+I & W | L+I | 3.90 | 0.041 |
| Weissella | L+I & W | L+I | 4.57 | 0.003 |
| Enterococcus | L+I & L+W | L+I | 3.52 | 0.026 |

**Table S7. Bacterial genera unique to each treatment.** Unique bacterial genera identified for each experimental treatment as defined in the UpSet plot analysis. No unique genera were identified for the Cell Wall (W) treatment.

| **Control** | ***Lactobacillus*** | **Inulin** | ***Lactobacillus* + Inulin** | ***Lactobacillus* + cell wall** |
| --- | --- | --- | --- | --- |
| *Anaerobacter* | *Azospirillum* | *Actinokineospora* | *Alcanivorax* | *Delftia* |
| *Bifidobacterium* | *Bosea* | *Enterobacter* | *Devosia* | *Enhydrobacter* |
| *Desulfomicrobium* | *Halodesulfovibrio* | *Filobacterium* | *Kocuria* | *Serratia* |
| *Exiguobacterium* | *Hydrogenobaculum* | *Leuconostoc* | *Lactococcus* |  |
| *Marinobacterium* | *Iphinoe* | *Maribacter* | *Ligilactobacillus* |  |
| *Rhodoligotrophos* | *Paracoccus* | *Micrococcus* |  |  |
| *Romboutsia* | *Reyranella* | *Nitriliruptor* |  |  |
| *Shewanella* | *Rhodobacter* | *Ruminococcus* |  |  |
|  | *Roseiflexus* |  |  |  |
|  | *Rubellimicrobium* |  |  |  |
|  | *Thermodesulfovibrio* |  |  |  |
|  | *Yoonia-Loktanella* |  |  |  |
